# Sequential Modulations of Tumor Vasculature and Stromal Barriers Augment the Active Targeting Efficacy of Antibody‐Modified Nanophotosensitizer in Desmoplastic Ovarian Carcinoma

**DOI:** 10.1002/advs.202002253

**Published:** 2020-12-23

**Authors:** Yue Yan, Binlong Chen, Zenghui Wang, Qingqing Yin, Yaoqi Wang, Fangjie Wan, Yulin Mo, Bo Xu, Qiang Zhang, Siling Wang, Yiguang Wang

**Affiliations:** ^1^ School of Pharmacy Shenyang Pharmaceutical University Shenyang Liaoning 110016 China; ^2^ Beijing Key Laboratory of Molecular Pharmaceutics and New Drug Delivery Systems School of Pharmaceutical Sciences Peking University Beijing 100191 China; ^3^ State Key Laboratory of Natural and Biomimetic Drugs Peking University Beijing 100191 China

**Keywords:** active tumor targeting, antibody‐modified nanophotosensitizer, nonspecific uptake, stromal barriers, tumor accumulation

## Abstract

Active‐targeted nanoparticles are attractive carriers due to their potentials to facilitate specific delivery of drugs into tumor cells while sparing normal cells. However, the therapeutic outcomes of active‐targeted nanomedicines are hampered by the multiple physiological barriers in the tumor microenvironment (TME). Herein, an epidermal growth factor receptor‐targeted ultra‐pH‐sensitive nanophotosensitizer is fabricated, and the regulation of the TME to augment the active targeting ability and therapeutic efficacy is pinpointed. The results reveal that tumor vasculature normalization with thalidomide indiscriminately enhance the tumor accumulation of passive and active targeted nanoparticles, both of which are sequestered in the stromal bed of tumor mass. Whereas, photoablation of stromal cells located in perivascular regions significantly improves the accessibility of antibody‐modified nanophotosensitizer to receptor‐overexpressed cancer cells. After sequential regulation of TME, the antitumor efficacy of antibody‐modified nanophotosensitizer is drastically enhanced through synergistic enhancements of tumor accumulation and cancer cell accessibility of active‐targeted nanoparticles. The study offers deep insights about the intratumoral barriers that hinder the active‐targeted nanoparticles delivery, and provides a basis for developing more effective strategies to accelerate the clinical translation of active‐targeted nanomedicines.

## Graphical abstract

Sequential regulations of tumor microenvironment by thalidomide and pre‐photodynamic therapy (pre‐PDT) treatments are exploited to achieve synergistic enhancements of tumor accumulation and cancer cell accessibility of epidermal growth factor receptor (EGFR)‐targeted micelle, thereby improve the targeting ability and therapeutic outcome of the active‐targeted nanoparticles.

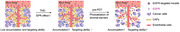

## 1 Introduction

Chemotherapy and radiotherapy have been clinically applied as the basic cancer therapy modalities for decades.^[^ ^1^ ^]^ However, it still remains a challenge to achieve effective cancer therapy due to the poor selectivity of chemotherapy and radiotherapy.^[^ ^2^ ^]^ Thus, there is an urgent need to develop more effective and specific treatment strategies. Cancer is known as a heterogeneous disease that develops via a multistep process.^[^ ^3^ ^]^ During the process, cancer adopts various hallmarks that different from normal tissues, such as acidic pH levels, reductive conditions, and overexpression of certain cell membrane receptors.^[^ ^4^ ^]^ Hence, stimuli‐responsive drug delivery systems and tumor‐specific antibodies have been developed for improved tumor theranostics that benefit greatly from the differences between tumors and healthy tissues. Active‐targeted therapy that combines nanoparticles with tumor‐recognized antibodies stands out as a highly specific cancer treatment.^[^ ^5^ ^]^ It can achieve selective drug delivery to cancer cells while sparing normal cells. Because of the superior tumor‐targeting ability in vitro, the investigation on active‐targeted nanoparticles has shown a steep rise in the last decade.^[^ ^6^ ^]^ However, only a few active‐targeted nanoparticles are undergoing clinical trials and none of them has been approved for clinical applications due to their mediocre therapeutic outcomes in vivo.^[^ ^7^ ^]^

The enough delivery of nanoparticles in the tumor site is the prerequisite of superior therapeutic effect. However, it is reported that only about 0.7% of intravenously administered nanoparticles can be delivered to solid tumors through electron paramagnetic resonance (EPR) effect.^[^ ^8^ ^]^ Moreover, due to the dense extracellular matrix,^[^ ^9^ ^]^ nonspecific interaction with stromal cells,^[^ ^10^ ^]^ and high intratumoral interstitial pressure in the tumor microenvironment,^[^ ^11^ ^]^ a majority of active‐targeted nanoparticles are trapped in the perivascular regions and fail to directly interact with tumor cells overexpressed with specific receptors.^[^ ^12^ ^]^ These pathophysiological barriers in solid tumor further contributes to the poor therapeutic effect of nanomedicine.^[^ ^13^ ^]^ Therefore, both poor tumor accumulation and off‐target effect are responsible for the limited targeting abilities and therapeutic outcomes of active‐targeted nanoparticles.

Benefiting from the minimal invasion and fast healing, photodynamic therapy (PDT) has been approved by the Food and Drug Administration (FDA) for cancer treatments.^[^ ^14^ ^]^ Nanoparticle‐based delivery strategy offers special benefits for PDT due to the well‐known EPR effect.^[^ ^15^ ^]^ However, most of nanophotosensitizers are always‐ON systems with inevitable distribution in healthy tissues, leading to long‐lasting phototoxicity.^[^ ^16^ ^]^ To overcome this drawback, activatable PDT which can turn OFF the photosensitization in healthy tissues, while turn ON by specific stimuli in tumors has been developed.^[^ ^17^ ^]^ In addition, active‐targeted PDT has also been exploited to further increase the tumor specificity of nanophotosensitizers.^[^ ^18^ ^]^ Nevertheless, the in vivo tumor targeting and therapeutic efficacy of active‐targeted nanophotosensitizers are still unsatisfactory and ambiguous.^[^ ^19^ ^]^

**Scheme 1:**
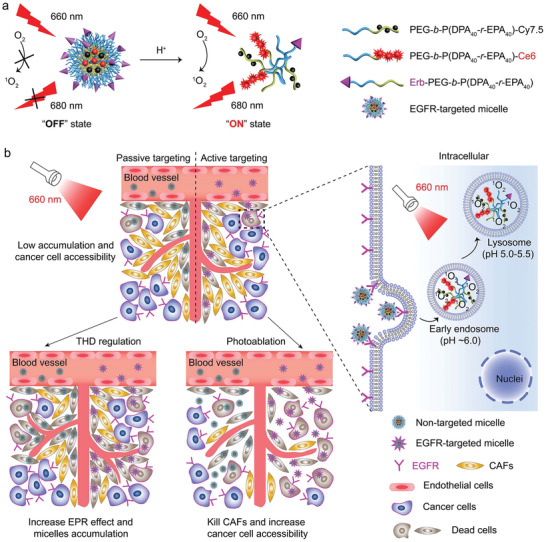
Schematic illustration of the delivery of acid‐activatable EGFR‐targeted nanophotosensitizer after modulations of tumor microenvironment. a) pH‐modulated Ce6 fluorescence activation and singlet oxygen generation of the EGFR‐targeted ultra‐pH‐sensitive photodynamic micelle. b) Left panel: Modulating the tumor microenvironment by thalidomide for enhanced tumor accumulation and photoablation of stromal cells to improve the in vivo PDT therapeutic outcomes of EGFR‐targeted nanophotosensitizers. Right panel: The EGFR‐targeted micelles are specifically internalized via EGFR‐mediated endocytosis, dissociate in the acidic endocytic organelles, and generate singlet oxygen under 660 nm irradiation to kill the tumor cells.

Herein, we developed an epidermal growth factor receptor (EGFR) targeted ultra‐pH‐sensitive nanophotosensitizer, and systemically investigated its active targeting ability and therapeutic efficacy after the regulation of tumor vasculature and stromal barriers. EGFR, as one of the most common targets for cancer therapy, is reported to be overexpressed on the surface of a wide variety of cancer cells.^[^ ^20^ ^]^ The Fab’ fragment of Erbitux, a clinically applied antibody of EGFR, was conjugated to the nanophotosensitizers as the targeting moiety. Our study verified that the antibody‐modified nanophotosensitizers showed specific targeting ability and superior cytotoxicity in vitro compared with their nontargeted counterparts. However, the in vivo tumor targeting and inhibition efficacy were compromised due to the poor accumulation and nonspecific sequestration by stromal cells. Then, the tumor microenvironment was perturbed to augment the in vivo behaviors of antibody‐modified nanophotosensitizers. First, enhancing the EPR effect through vasculature normalization with thalidomide (THD) treatment identically improved the tumor accumulation and antitumor efficacy of passive and active‐targeted nanophotosensitizers without significant improvement on active targeting ability. Second, both the tumor cell‐targeting ability and therapeutic efficacy of antibody‐modified nanophotosensitizers dramatically increased after photoablating the stromal cells by pre‐PDT treatment. Finally, we conducted the regulation of tumor microenvironment by sequential THD and pre‐PDT treatments to realize synergistic enhancements on tumor accumulation and targeting ability of antibody‐modified nanophotosensitizers, further highlighting the superiority of active‐targeted strategy (**Scheme** **1**). Overall, this regulation strategy holds great promise for actively targeted delivery of therapeutic nanoparticles and enhanced antitumor efficacy.

## 2 Results and Discussion

### 2.1 Synthesis and Characterization of EGFR‐Targeted Acid‐Activatable Nanophotosensitizer

**Figure 1:**
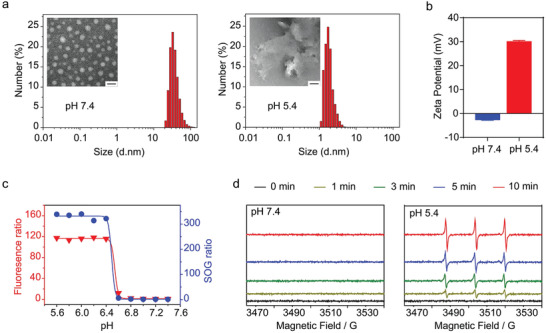
Characterizations of the pH‐triggered fluorescence and SOG activation of Erb‐AAPS. a) The particle size distribution and TEM images of Erb‐AAPS at pH 7.4 and pH 5.4, respectively. Scale bar = 50 nm. b) Zeta potentials of Erb‐AAPS at pH 7.4 and pH 5.4. Data were presented as mean ± s.d. (*n* = 3). c) The Ce6 fluorescence ratio and SOG ratio (F_pH_ / F_pH 7.4_) of Erb‐AAPS as a function of pH (Ce6 concentration, 10 µg mL^−1^). The SOG was estimated by p‐nitrosodimethylaniline (RNO) method with 660 nm irradiation at 100 mW cm^−2^ for 2 min. d) The SOG of Erb‐AAPS detected by EPR spectroscopy when irradiated with 660 nm laser at 100 mW cm^−2^ for different time.

The long‐lasting phototoxicity caused by inevitable distribution of photosensitizer (PS) in skin and eyes remains a major challenge for PDT.^[^ ^21^ ^]^ To overcome this drawback, we fabricated an acid‐activatable nanophotosensitizer, which could shut down the photosensitization of PS in healthy tissues, while fast switch on in the acidic endosome/lysosome of targeted cells after endocytosis. First, the ultra‐pH‐sensitive polymers PEG‐*b*‐P(DPA‐*r*‐EPA) with and without maleimide group were synthesized by atom transfer radical polymerization method as reported previously,^[^ ^22^ ^]^ and conjugated with photosensitizer Ce6‐COOH for photodynamic therapy. Cy7.5‐NHS and Cy5‐NHS esters were also conjugated to the polymers as fluorescence quencher and in vivo imaging probe, respectively (Table S1 and Figure S1, Supporting Information). The nontargeted acid‐activatable nanophotosensitizer (AAPS) consisting of Ce6‐ and Cy7.5‐conjugated polymers at a molar ratio of 1: 1, was prepared using the sonication method as previously published.^[^ ^23^ ^]^ For the preparation of antibody‐modified nanophotosensitizer, the Fab’ fragments were first obtained via Erbitux enzymolysis followed by dithiothreitol (DTT) reduction. Then the 5% maleimide‐terminated AAPS was functionalized with Fab’ fragments to obtain the EGFR‐targeted acid‐activatable nanophotosensitizer (Erb‐AAPS) (Figures S2 and S3, Supporting Information). Both the AAPS and Erb‐AAPS were monodispersed spherical nanoparticles with diameters of 29.5 ± 1.8 and 34.5 ± 2.3 nm at pH 7.4, respectively. The particle sizes of these two nanoparticles decreased to below 5 nm at pH 5.4 due to the acid‐induced micelle dissociation (**Figure** **1**a; and Figure S4a,b, Supporting Information). The Fab’ modification exhibited negligible influence on the fluorescence properties and zeta potentials of micelles (Figure 1b; and Figure S4c,d, Supporting Information).

We then investigated the acid‐activatable fluorescence and singlet oxygen generation (SOG) of AAPS and Erb‐AAPS. As shown in Figure 1c; and Figure S5 in the Supporting Information, both AAPS and Erb‐AAPS exhibited sharp pH response and high fluorescence (>100 folds) and SOG (>300 folds) activation ratios with a pH transition (pH_t_) of ≈6.5. Besides, the pH‐induced SOG activation displayed perfect correlation with Ce6 fluorescence activation curve (Figure 1c; and Figure S6a, Supporting Information). To further verify the acid‐induced SOG amplification of nanophotosensitizers, the generated singlet oxygen at different pH was also detected by EPR method. Results indicated that there was no detectable SOG signal under abundant 660 nm irradiation at pH 7.4. In contrast, vigorous SOG was visualized at pH 5.4 over irradiation time (Figure 1d; and Figure S6b, Supporting Information), confirming the pH‐triggered SOG abilities of nanophotosensitizers. In addition, the AAPS and Erb‐AAPS kept stable in serum over 24 h, as evidenced by the negligible changes of Ce6 fluorescence and SOG (Figure S7, Supporting Information). Therefore, we successfully established the nontargeted AAPS and EGFR‐targeted Erb‐AAPS, both of which exhibited two orders of magnitude amplification for fluorescence and SOG signals in response to pH changes. The photosensitization of Ce6 was quenched at physiological pH due to the FRET effect between Ce6 and Cy7.5. However, the micelles quickly dissociated into unimers when pH < pH_t_, accompanied with the activation of fluorescence and SOG (under 660 nm irradiation).

### 2.2 In Vitro Targeting and Photokilling Efficacy of Erb‐AAPS

**Figure 2:**
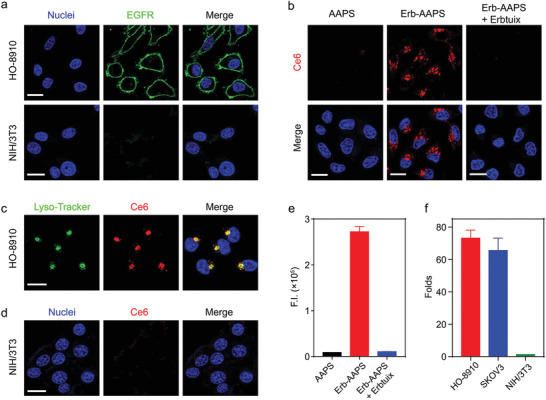
In vitro EGFR‐positive cancer cell‐targeting of Erb‐AAPS. a) Immunohistochemical staining of EGFR expression on HO‐8910 ovarian cancer cells and NIH/3T3 fibroblasts. b) The cellular uptake of AAPS and Erb‐AAPS (Ce6 concentration, 10 µg mL^−1^) in HO‐8910 cells after incubation at 37°C for 4 h. For competitive assay, the cells were pre‐treated with 10 µg mL^−1^ Erbitux for 1 h, and then incubated with Erb‐AAPS in the presence of excessive Erbitux. Red: Ce6. Blue: nuclei. c) Confocal images of intracellular colocalization of Erb‐AAPS with lysosomes. Lysotracker Green was used to label the lysosomes. d) Confocal images of the cellular uptake of Erb‐AAPS in EGFR‐negative NIH/3T3 cells. e) Quantitative cellular uptake of AAPS and Erb‐AAPS in HO‐8910 cells analyzed by flow cytometry (*n* = 3). f) The targeting efficacy of Erb‐AAPS in different cell lines after incubation at 37°C for 4 h (*n* = 3). All data were presented as mean ± s.d. Scale bars = 20 µm.

EGFR is one of the important targets for active‐targeted anticancer therapy.^[^ ^24^ ^]^ Here, we prepared the Erb‐AAPS by functionalizing the AAPS with the Fab’ fragments of Erbitux.^[^ ^25^ ^]^ To estimate the targeting ability of the Erb‐AAPS, we detected the EGFR expression levels of various cells at first. It was found that HO‐8910, SKOV3, and BxPC‐3 tumor cells expressed high levels of EGFR, while the NIH/3T3 fibroblasts were EGFR‐negative cells (**Figure** **2**a; and Figures S8–S10, Supporting Information). Therefore, EGFR‐positive HO‐8910 and SKOV3 cells were used to evaluate the targeting ability of Erb‐AAPS, whereas EGFR‐negative NIH/3T3 cell was applied as negative control. As shown in Figure 2b, the cellular uptake of Erb‐AAPS in HO‐8910 cells was dramatically higher than their nontargeted counterparts (AAPS) and their targeting abilities could be blocked by Erbitux antibody. The fluorescent signal of endocytosed Erb‐AAPS was perfectly colocalized with LysoTracker, demonstrating the pH‐activation of the active‐targeted nanophotosensitizer in endosomes/lysosomes (Figure 2c). In marked contrast, there was no appreciable uptake of Erb‐AAPS observed in NIH/3T3 cells without EGFR expression (Figure 2d). Next, the EGFR‐mediated uptake of Erb‐AAPS in HO‐8910 cells was further corroborated by flow cytometry (FCM) (Figure 2e). To verify the universality, the active targeting ability of Erb‐AAPS was also verified on SKOV3 cells (Figure S11, Supporting Information). Quantitative analyses indicated that the antibody‐modified Erb‐AAPS achieved over 70‐fold higher internalization than the passive‐targeted AAPS in both HO‐8910 and SKOV3 tumor cells (Figure 2f), while no significance between the uptake of the two nanophotosensitizers was observed in NIH/3T3 fibroblasts. Overall, these results indicated that Erb‐AAPS could target to EGFR‐positive tumor cells with high specificity.

**Figure 3:**
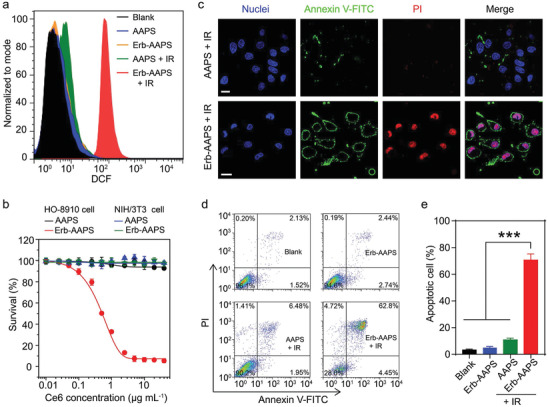
In vitro photodynamic efficacy of Erb‐AAPS. a) Intracellular SOG of AAPS and Erb‐AAPS (Ce6 concentration, 10 µg mL^−1^) in HO‐8910 cells with/without 660 nm irradiation, using DCFH‐DA as singlet oxygen indicator. b) The photodynamic effect of Erb‐AAPS in EGFR‐positive HO‐8910 cells and EGFR‐negative NIH/3T3 cells, respectively. The cells were incubated with AAPS or Erb‐AAPS (Ce6 concentration, 10 µg mL^−1^) for 4 h, followed by 660 nm irradiation at 100 mW cm^−2^ for 3 min. The cell survival was assessed at 24 h postirradiation by MTT assay. Data were presented as mean ± s.d. (*n* = 3). c) Confocal images, d) FCM analysis and e) Quantification of cell apoptosis induced by AAPS or Erb‐AAPS‐mediated PDT indicated with Annexin V and PI staining. Scale bars = 20 µm. Data were presented as mean ± s.d. (*n* = 3). Statistical analyses were performed by student's t‐test, ***p < 0.001.

Subsequently, we evaluated the in vitro photodynamic effect of Erb‐AAPS on HO‐8910 cells and NIH/3T3 cells, respectively. The intracellular singlet oxygen generated by nanophotosensitizers after irradiation was first detected by flow cytometry using 2′,7′‐dichlorfluorescein‐diacetate (DCFH‐DA) as singlet oxygen indicator. Results demonstrated that up to 24‐fold higher SOG was produced in Erb‐AAPS treated HO‐8910 cells as compared with AAPS treated cell group under 660 nm irradiation (**Figure** **3**a). Afterward, the cytotoxicity on HO‐8910 cells and NIH/3T3 cells with PDT treatment was evaluated by MTT (3‐(4,5‐dimethylthiazol‐2‐yl)‐2,5‐diphenyltetrazolium bromide) assay. The Erb‐AAPS (up to 20 µg mL^−1^ Ce6) exhibited negligible cytotoxicity after incubation with HO‐8910 cells in dark for 24 h (Figure S12a, Supporting Information), suggesting the excellent biosafety of Erb‐AAPS. After irradiation with 660 nm laser, the Erb‐AAPS showed a remarkably superior photodynamic efficacy as compared to nontargeted AAPS on EGFR‐positive HO‐8910 cells (Figure 3b), which could be attributed to the efficient uptake of Erb‐AAPS via the EGFR‐mediated endocytosis. Whereas both AAPS and Erb‐AAPS exhibited no obvious phototoxicity on NIH/3T3 cells, due to the low EGFR expression on NIH/3T3 cells. Subsequently, Annexin V‐FITC/PI staining was applied to investigate the cell death mechanism evoked by Erb‐AAPS‐mediated PDT. As shown in Figure 3c; and Figure S12b in the Supporting Information, Erb‐AAPS induced vigorous apoptosis upon 660 nm irradiation both on HO‐8910 cells and SKOV3 cells, while neither apoptosis nor necrosis was induced by AAPS. Quantitative results determined by FCM further confirmed that Erb‐AAPS provoked 62.8% apoptosis with 660 nm irradiation, 10‐fold higher than that induced by AAPS (Figure 3d,e). Taken together, these results confirmed that the Erb‐AAPS could specifically recognize the EGFR‐overexpressing cancer cells, resulting in significantly enhanced cellular uptake and PDT efficacy.

### 2.3 Poor Accumulation and Stromal Cells Sequestration Weaken the In Vivo Tumor Targeting and Therapeutic Efficacy of Erb‐AAPS

**Figure 4:**
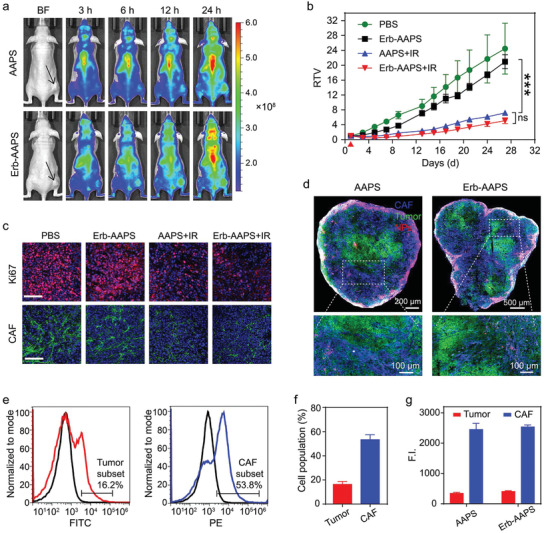
Poor accumulation and CAFs sequestration weaken the in vivo tumor targeting and therapeutic efficacy of Erb‐AAPS. a) In vivo fluorescence images of the HO‐8910 tumor‐bearing mice at 3, 6, 12, and 24 h post‐injection of micelles. The tumor regions were indicated by the black arrows. b) Relative tumor volume (RTV) changes of subcutaneous HO‐8910 tumors in mice with different treatments. For IR groups, the tumors of mice administrated with AAPS or Erb‐AAPS micelles (Ce6 dose of 3 mg kg^−1^) were subjected to 660 nm irradiation at 400 mW cm^−2^ for 10 min at 3 h post‐injection. The red triangle represents the time point for PDT treatment. Data were presented as mean ± s.d. (*n* = 5). Statistical analyses were performed by two‐way ANOVA with Tukey's post‐hoc test, ***p < 0.001, ns = not significant. c) Immunofluorescence staining of proliferating cells and CAFs in tumor tissues at 36 h post‐treatment. Scale bars = 100 µm. d) The distribution of AAPS and Erb‐AAPS nanoparticles (NPs) in the tumor tissues. CAF, HO‐8910 and NPs were denoted as blue, green, and red signals, respectively. e) Flow cytometry and f) quantitative results (mean ± s.d., *n* = 3) of the percentages of tumor cells and CAFs in HO‐8910 tumor tissues, respectively. g) The quantitative results for the amounts of AAPS and Erb‐AAPS endocytosed by tumor cells and CAFs, respectively (*n* = 3). Data were presented as mean ± s.d.

For in vivo imaging studies, Cy5 probe with superior fluorescence properties than Ce6 was applied to indicate the in vivo distribution of AAPS and Erb‐AAPS. A xenograft model of human ovarian cancer with poor EPR effect was selected in this study to investigate effects of the different TME regulations on the active targeting ability and therapeutic efficacy of active‐targeted micelles. As seen in **Figure** **4**a; and Figure S13 in the Supporting Information, after injected intravenously into the mice, the accumulation of AAPS and Erb‐AAPS at tumor sites increased in the first 3 days due to the long circulation time of micelles, then the micelles gradually cleared from tumor sites at a much slower rate than that of other body parts. Unfortunately, both AAPS and Erb‐AAPS showed low tumor accumulation due to the poor EPR effect of HO‐8910 tumor model. Moreover, unlike the superior targeting efficacy in vitro, Erbitux modification only achieved a slightly higher tumor accumulation in vivo without any significant difference. More importantly, in vivo antitumor study further revealed that AAPS and Erb‐AAPS achieved similar tumor inhibition efficacy with the same irradiation dose over a time course of 27 days (Figure 4b; and Figure S14, Supporting Information). The Ki67 staining also verified the comparable antiproliferation for the two nanophotosensitizers (Figure 4c). Interestingly, we found that the density of cancer associated fibroblasts (CAFs) was significantly reduced after PDT treatments, indicating that micelles could be endocytosed by CAFs (Figure 4c).

To elucidate the failure of the antibody‐modified micelles in vivo, the tumors were excised and sectioned at 24 h postinjection for immunofluorescence staining of tumor cells (EGFR) and stromal cells (ER‐TR7, CAFs marker). Results indicated that both AAPS and Erb‐AAPS nanoparticles (NPs) exhibited peritumoral distribution with poor tumor accumulation. In addition, these two NPs were mainly endocytosed by CAFs, and rarely reached the tumor cells, as evidence by the large number of purple signals and very few yellow signals in the tumor slices (Figure 4d). Moreover, we found that the HO‐8910 tumor presented a stroma‐vessel architecture that the CAFs located surrounding the tumor blood vessels (Figure S15, Supporting Information), which was in compliance with previous study.^[^ ^26^ ^]^ Afterward, flow cytometry was carried out to quantify the percentages of tumor cells and CAFs populations in the HO‐8910 tumors as well as the amounts of AAPS and Erb‐AAPS endocytosed by tumor cells and CAFs, respectively. As shown in Figure 4e,f, the tumor cells and CAFs accounted for 16.2% and 53.8% of the cell population in HO‐8910 tumor tissues, respectively. More surprisingly, both the nontargeted and EGFR‐targeted micelles were mainly internalized by CAFs, which were 7.2‐fold and 6.2‐fold higher than that endocytosed by tumor cells on a per cell population basis, respectively (Figure 4g). The quantitative results were in accordance with the qualitative immunofluorescence results, which are also consistent with previous studies.^[^ ^8^, ^10^, ^26^ ^]^ Hence, the spatial localization and abundance of CAFs in tumors resulted in the in vivo off‐target and failure of the Erbitux‐modified nanocarriers.

It has been reported that the nonspecific interaction with stromal cells including CAFs and macrophages could prevent the deep penetration of nanoparticles into solid tumors.^[^ ^10^ ^]^ In our study, CAFs was selected as a representative of stromal cells because they were rich in subcutaneous HO‐8910 tumor model. The above results showed that the antibody‐modified micelles achieved excellent targeting and therapeutic abilities in vitro, while exhibited unsatisfactory efficacy in vivo, which was due, at least partially, to the poor accumulation in tumor tissues and the nonspecific uptake by CAFs. The poor accessibility of antibody‐modified micelles to tumor cells was proved to be a great obstacle to exerting their therapeutic effects. Therefore, the antibody‐modified micelles failed to highlight their advantages, exhibiting the similar in vivo therapeutic effects as their nontargeted counterparts. Conceivably, improving tumor accumulation and eradicating CAFs would be two substantial strategies to increase the targeting abilities and therapeutic effects of antibody‐modified micelles.

### 2.4 Vasculature Normalization with Thalidomide Improves the Tumor Accumulation of Micelles

**Figure 5:**
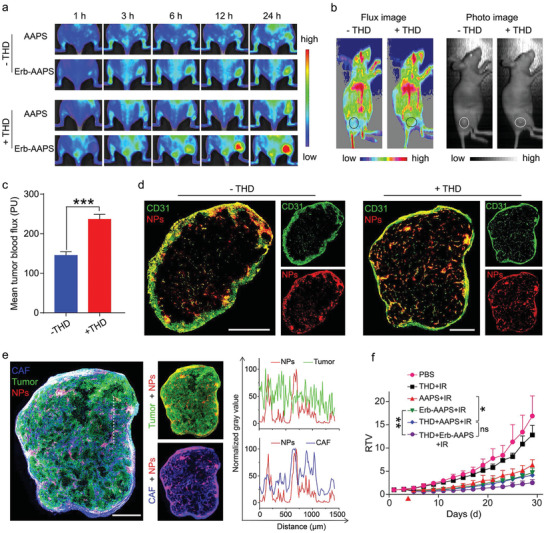
Vasculature normalization with thalidomide improves the tumor accumulation of micelles. a) In vivo fluorescence images of the HO‐8910 tumor‐bearing mice with or without THD treatment at 3, 6, 12, and 24 h post‐injection of micelles. The mice of THD regulation groups were orally treated with THD (50 mg kg^−1^) for three consecutive days before micelles administration. b) Images and c) quantitative results of tumor blood flow in HO‐8910 tumors with or without THD treatment measured by Laser Doppler imaging system. Tumor regions were marked by circles in flux images and photo images. Data were presented as mean ± s.d. (*n* = 4). Statistical analyses were performed by student's t‐test, ***p < 0.001. d) The vessel density and Erb‐AAPS NPs distribution in HO‐8910 tumors with or without THD treatment. Tumor vessel and NPs were denoted as green and red signals, respectively. e) Left panel: The distributions of CAFs, tumor cells and Erb‐AAPS NPs in THD‐treated HO‐8910 tumors. Middle panel: the merged fluorescent images of Erb‐AAPS with tumor cells and CAFs, respectively. CAF, HO‐8910 and NPs were denoted as blue, green, and red signals, respectively. Right panel: The extent of colocalization between Erb‐AAPS NPs and tumor cells (or CAFs) along the white dotted line in tumor slices of left panel. The data were analyzed by the Image J software. f) Relative tumor volume changes of subcutaneous HO‐8910 tumors in mice with different treatments. The mice of THD regulation groups were orally treated with THD for three consecutive days before the PDT treatment on day 4. Data were presented as mean ± s.d. (*n* = 5). Statistical analyses were performed by two‐way ANOVA with Tukey's post‐hoc test, *p < 0.05, **p < 0.01, ***p < 0.001, ns = not significant. Scale bars = 800 µm.

We then investigated whether improving the tumor accumulation of micelles could augment the in vivo efficacy of antibody‐modified micelles. THD has been previously used for both vessel normalization and angiogenesis inhibition. Recent studies revealed that a low dose of THD could increase the EPR effect to improve the accumulation of nanoparticles.^[^ ^27^ ^]^ Therefore, we here adopted a low‐dose thalidomide to remodel the tumor vasculature and increase the tumor accumulation of the micelles. According to the in vivo fluorescence imaging results in **Figure** **5**a, the accumulation of AAPS and Erb‐AAPS at tumor sites were both increased after THD treatment. Although the EGFR‐targeted micelles achieved higher tumor‐targeting efficacy than the nontargeted ones after THD treatment, there was no significant difference in comparison with non‐THD treatment (Figure S16, Supporting Information). Laser Doppler imaging results showed that the tumor blood flow increased in THD treated mice (Figure 5b,c; and Figure S17, Supporting Information), which could be a reason for the improved tumor accumulation.

Subsequently, the tumors were excised and sectioned at 24 h post‐injection. Immunofluorescence results showed that the tumor vessel density significantly increased after THD treatment, resulting in the enhanced micelle accumulation at the tumor sites (Figure 5d; and Figure S18, Supporting Information). However, the Erb‐AAPS still displayed low cancer cell accessibility and mostly endocytosed by CAFs after THD treatment (Figure 5e).

Next, we evaluated the photodynamic efficacy of micelles after THD regulation. The tumor inhibition of THD treatment groups were highly improved as compared to their non‐THD treatment counterparts (Figure 5f). However, the EGFR‐targeted micelles still failed to achieve a significant superior antitumor efficacy than the nontargeted ones after THD treatment. Collectively, THD regulation significantly increased the tumor vessel density and tumor blood flow, resulting in the enhanced accumulation of micelles at the tumor site and partially improved photodynamic therapy. Nevertheless, the majority of the antibody‐modified micelles were still nonspecifically internalized by CAFs, leading to the inconspicuous improvement in their accessibility to tumor cells, and the nonsignificant enhancement of the in vivo targeting ability.

### 2.5 Photoablation of CAFs by Pre‐PDT Regulation Enhances the Tumor Cells Accessibility

Since more than six‐fold nanoparticles were internalized by CAFs than tumor cells in vivo, we next investigated whether eradication of CAFs could improve the in vivo tumor targeting and therapeutic efficacy of antibody‐modified nanophotosensitizers. First of all, it is necessary to figure out why a majority of nanoparticles were internalized by CAFs in vivo. To mimic the interaction of tumor cells and CAFs in tumor microenvironment in vivo, we co‐cultured the fibroblasts with tumor cells for 48 h to acquire CAFs phenotype. Results indicated that the uptake capacity of activated fibroblasts was slightly higher than that of normal fibroblasts (Figure S19, Supporting Information). Additionally, CAFs have a high probability of interaction with active targeted nanoparticles extravasated from tumor vasculature due to its high abundance around perivascular regions, collectively leading to their high uptake of antibody‐modified micelles in vivo.

**Figure 6:**
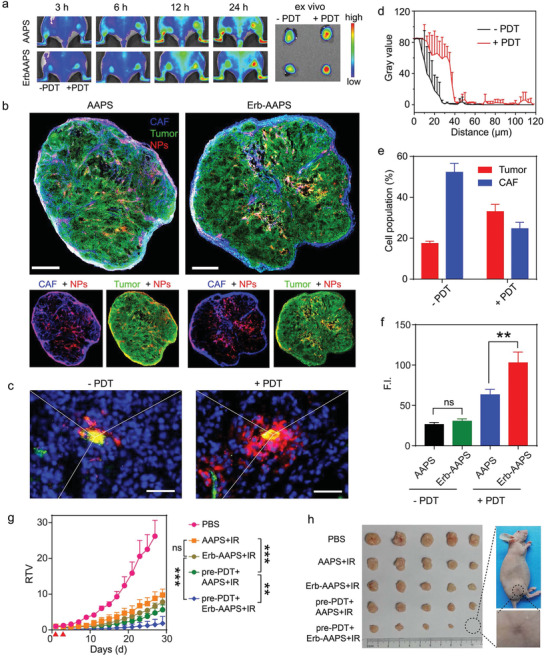
Photoablation of CAFs by pre‐PDT regulation enhances the tumor cells accessibility. a) Fluorescence images of bilateral HO‐8910 tumor‐bearing mice with or without pre‐PDT regulation at 3, 6, 12, and 24 h post‐injection. The pre‐PDT treatment was performed on the right tumors at 36 h before micelles administration. b) The colocalization analysis of the AAPS and Erb‐AAPS NPs with tumor cells and CAFs in the pre‐PDT treated tumors, respectively. CAF, HO‐8910, and NPs were denoted as blue, green, and red signals, respectively. Scale bar = 800 µm. c) Diffusion of antibody‐modified micelles from tumor vessels into the distal regions in tumors with and without PDT treatment. Scale bar = 50 µm. d) Quantitative results of Figure 6c by Image J software (mean ± s.d., *n* = 3). e) Quantitative proportion of tumor cells and CAFs before and after pre‐PDT treatments by flow cytometry (mean ± s.d., *n* = 3). f) Quantitative amounts of nanoparticles endocytosed by tumor cells before and after pre‐PDT treatment, respectively. Data were presented as mean ± s.d. (*n* = 4). Statistical analyses were performed by student's t‐test, **p < 0.01, ns = not significant. g) Growth curves for tumors of HO‐8910 tumor‐bearing mice with different treatments. The pre‐PDT treatment was given at day 1 and subsequent PDT treatment was given at day 3, as indicated by the red triangle. h) The photographic images of excised tumors at the end of anti‐tumor study. Data were presented as mean ± s.d. (*n* = 5). Statistical analyses were performed by two‐way ANOVA with Tukey's post‐hoc test, *p < 0.05, **p < 0.01, ***p < 0.001, ns = not significant.

Inspired by the results that considerable amounts of CAFs were ablated by AAPS‐mediated PDT, we employed a pre‐PDT treatment for CAFs removal to enhance the accessibility of micelles to tumor cells. The bilateral HO‐8910 tumor‐bearing mice were intravenously injected with AAPS, then the right tumors of the mice were irradiated with 660 nm laser at 3 h postinjection for pre‐PDT regulation. As shown in **Figure** **6**a, both the nontargeted and antibody‐modified micelles exhibited more accumulation in the pre‐PDT (right) tumors, probably due to the enhanced tumor permeability induced by CAFs eradication. In addition, the active‐tumor targeting efficacy of the EGFR‐targeted micelles was significantly improved after pre‐PDT treatment (Figure S20, Supporting Information). Next, the increased tumor accumulation of micelles was also verified by the immunofluorescence staining study (Figure 6b). Besides, the accessibility of EGFR‐targeted micelles to tumor cells was also increased after pre‐PDT regulation, as indicated by the better colocalization (yellow color) between Erb‐AAPS NPs and EGFR in the tumor slices (Figure S21, Supporting Information).

We next investigated the penetration of micelles into tumor tissues after pre‐PDT treatment. As seen in Figure 6c, the antibody‐modified micelles mainly distributed within the vessel lumen and perivascular regions before PDT treatment, while penetrated deep into regions away from tumor blood vessels after PDT treatment. The effective diffusion distance of micelles in PDT treated tumor could be 40 µm, fourfold longer than that in control tumor (Figure 6d). These results confirmed that the pre‐PDT treatment could effectively remove CAFs around the tumor blood vessels, and enhance the extravasation of Erb‐AAPS from tumor blood vessels into the deep tumor regions, thereby increase the cancer cell accessibility of Erb‐AAPS.

Subsequently, flow cytometry further quantitatively revealed that the proportion of CAFs was considerably decreased from 52.3% to 24.7% after PDT treatment, accompanied with the increased proportion of tumor cells from 17.5% to 33.0% (Figure 6e, Figure S22, Supporting Information). As a result, the antibody‐modified micelles trapped by CAFs were significantly reduced, while their accessibility to EGFR‐overexpressing tumor cells were significantly improved. More strikingly, the amount of antibody‐modified micelles internalized by tumor cells after PDT treatment was significantly enhanced as compared with that of nontargeted ones, suggesting the notably enhanced active targeting delivery of the antibody‐modified micelles into tumor cells (Figure 6f; and Figure S23, Supporting Information).

We further evaluated the in vivo photodynamic effect of micelles after pre‐PDT regulation. The tumor inhibition effect of pre‐PDT treatment groups was highly improved as compared to their nontreatment counterparts (Figure 6g). Moreover, the antibody‐modified micelles achieved a highly superior antitumor effect than the nontargeted ones after pre‐PDT regulation, with one tumor completely removed (Figure 6h; and Figure S24, Supporting Information). Taken together, eradicating CAFs by pre‐PDT treatment significantly increased the diffusion distances of antibody‐modified micelles and enhanced the accessibility to cancer cells, resulting in the significant enhancement of the targeting ability and antitumor efficacy of antibody‐modified micelles.

### 2.6 Combined Tumor Regulations Synergistically Amplified Active Targeting and Therapeutic Efficacy

**Figure 7:**
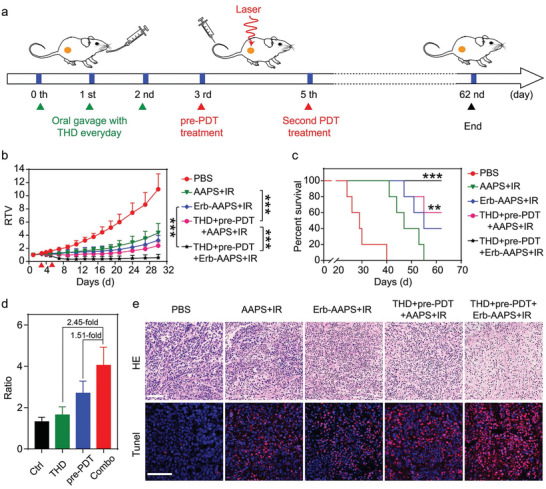
Combined tumor regulations synergistically amplified active targeting and therapeutic efficacy. a) Therapeutic schedule for active‐targeted PDT combined with THD and pre‐PDT regulations. For combined THD and pre‐PDT regulation groups, the mice were orally treated with THD for three consecutive days on day 0, 1 and 2, followed by pre‐PDT regulation on day 3, and then subjected to the second PDT treatment at day 5 by 660 nm irradiation at 400 mW cm^−2^ for 10 min at 3 h post‐injection of AAPS or Erb‐AAPS (Ce6 dose of 3 mg kg^−1^). b) Relative tumor volume changes of subcutaneous HO‐8910 tumors in mice with or without combined tumor regulations. The red triangle represents the time point for PDT treatment. Data were presented as mean ± s.d. (*n* = 5). Statistical analyses were performed by two‐way ANOVA with Tukey's post‐hoc test, *p < 0.05, **p < 0.01, ***p < 0.001, ns = not significant. c) The survival curves of mice in b). (Statistical analyses were performed using a log‐rank test, *n* = 5, **p < 0.01, ***p < 0.001 versus PBS group). d) The ratios of residual tumor volumes between AAPS and Erb‐AAPS groups after different regulation methods at day 27 (*n* = 5). e) H&E and TUNEL staining of tumor sections collected at the 2 days post second PDT treatment. Scale bars = 100 µm. All data were presented as mean ± s.d. *p < 0.05, **p < 0.01, ***p < 0.001.

Considering that vasculature normalization by THD treatment and photoablation of stromal cells by pre‐PDT treatment could individually augment in vivo tumor accumulation and active targeting of antibody‐modified micelles, we finally investigated whether sequential THD and pre‐PDT treatment could further amplify the in vivo targeting and photodynamic efficacy of antibody‐modified micelles (**Figure** **7**a). Results in Figure 7b showed that the regulation groups exhibited remarkably better tumor inhibition than nonregulation groups. In addition, compared with AAPS, Erb‐AAPS achieved strikingly superior photodynamic efficacy after sequential regulations with two tumors completely removed (Figure S25, Supporting Information). The mice survival study agreed well with the tumor growth profiles. The mice of THD+pre‐PDT+Erb‐AAPS+IR group demonstrated the longest lifespan in comparison with other four groups (Figure 7c). More importantly, the superiority of Erb‐AAPS after combined regulation was 2.45‐fold and 1.51‐fold more significant than mono‐THD and mono‐pre‐PDT regulation, respectively, indicating the vigorously amplified targeting ability of antibody‐modified nanophotosensitizers (Figure 7d). The synergistic effect of THD and pre‐PDT regulations on promoting the efficacy of Erb‐AAPS was further verified by the H&E (hematoxylin and eosin) and TUNEL (Terminal deoxynucleotidyl transferase dUTP nick end labeling) staining of tumor slides (Figure 7e). Meanwhile, there was no significant damage to the main organs as demonstrated by H&E staining (Figure S26, Supporting Information). Furthermore, blood routine examination revealed that all groups exhibited no distinct toxicity to blood cells (Figure S27, Supporting Information). All above data suggest the good biosafety of AAPS and Erb‐AAPS due to the pH‐activatable design.

In recent years, many studies have been devoted to investigate the remodeling of tumor barriers to improve the tumor penetration and therapeutic effects of nontargeted nanoparticles.^[^ ^10^, ^28^ ^]^ In addition, only a few researches have been conducted to clarify the failure of the tumor cell targeting for active‐targeted nanoparticles in vivo.^[^ ^8^, ^26^ ^]^ However, there are no systematic studies focusing on TME regulation to enhance the tumor cell targeting of active‐targeted nanoparticles. To this end, we synthesized an antibody‐modified pH‐activatable polymeric nanophotosensitizer, and systematically explored what caused the low tumor cell targeting efficacy of active‐targeted nanoparticles, how to improve it, and the correlation between tumor cell targeting ability and antitumor efficacy. First, THD regulation was used for tumor vasculature normalization to improve the tumor accumulation of nanophotosensitizers. This strategy identically enhanced the tumor accumulation and antitumor efficacy of passive‐ and active‐targeted nanophotosensitizers without significant improvement on specific targeting delivery to tumor cells. Then, “two hits” of PDT were exploited to achieve the enhanced antitumor efficacy of active‐targeted nanoparticle. The first hit (pre‐PDT regulation) was utilized to efficiently photoablate the stromal barriers (e.g., CAFs) at perivascular areas, allowing the deep penetration of nanoparticles into tumor sites and enhanced tumor cell targeting efficiency of active‐targeted nanoparticles. The second hit was employed to achieve photokilling of tumor cells after the specific delivery of active‐targeted nanophotosensitizers into target cells. Thus, when applying the sequential TME regulation strategy, active‐targeted nanophotosensitizers achieved significantly augmented tumor cell targeting efficiency and antitumor efficacy in desmoplastic ovarian carcinoma.

## 3 Conclusions

In summary, we demonstrated that tumor vasculature normalization through thalidomide regulation significantly augmented the tumor accumulation of intravenously administrated nanoparticles, which is the prerequisite for the nanoparticles targeting to specific tumor cells. After photoablating the stromal barriers at perivascular areas, the extravasated nanoparticles could penetrate into the deep regions of the tumor tissues, allowing for the direct interaction of active‐targeted nanoparticles with cancer cells. Combining these two regulation strategies, the antitumor efficacy was successfully augmented through synergistic enhancements of tumor accumulation and cancer cell accessibility of active‐targeted nanoparticles. The sequential regulation of TME represents an efficient and promising strategy toward actively targeted delivery of cancer therapeutics.

## 4 Experimental Section

4.1

4.1.1

4.1.1.1

Materials and experimental details are provided in the Supporting Information.

All animal procedures were performed according to the Guidelines approved by the Institutional Animal Care and Use Committee (IACUC) of Peking University (Accreditation number: LA2019039).

## Conflict of Interest

The authors declare no conflict of interest.

## Supporting information

Supporting Information
