# Supplementary material for: Sequential Modulations of Tumor Vasculature and Stromal Barriers Augment the Active Targeting Efficacy of Antibody‐Modified Nanophotosensitizer in Desmoplastic Ovarian Carcinoma
Source: Adv Sci (Weinh). 2020 Dec 23;8(3):2002253. doi: 10.1002/advs.202002253 (PMC7856881; doi:10.1002/advs.202002253)
Supplement: Supplementary file 1 — Supporting Information [file ADVS-8-2002253-s001.pdf]

WILEY-VCH

© 2020 Wiley-VCH GmbH

ADVANCED SCIENCE
Open Access

Supporting Information

for *Adv. Sci.,* DOI: 10.1002/advs.202002253

Sequential Modulations of Tumor Vasculature and Stromal Barriers Augment the Active Targeting Efficacy of Antibody-Modified Nanophotosensitizer in Desmoplastic Ovarian Carcinoma

Yue Yan, Binlong Chen, Zenghui Wang, Qingqing Yin, Yaoqi Wang, Fangjie Wan, Yulin Mo, Bo Xu, Qiang Zhang, Siling Wang, Yiguang Wang**

© 2020 Wiley-VCH GmbH

Supporting Information

Sequential Modulations of Tumor Vasculature and Stromal Barriers Augment the Active Targeting Efficacy of Antibody-Modified Nanophotosensitizer in Desmoplastic Ovarian Carcinoma

Yue Yan, Binlong Chen, Zenghui Wang, Qingqing Yin, Yaoqi Wang, Fangjie Wan, Yulin Mo, Bo Xu, Qiang Zhang, Siling Wang, Yiguang Wang**

Y. Yan, Prof. S. Wang
School of Pharmacy
Shenyang Pharmaceutical University
Shenyang, Liaoning 110016, China.
Email: silingwang@syphu.edu.cn

Y. Yan, Dr. B. Chen, Z. Wang, Q. Yin, Y. Wang, F. Wan, Y. Mo, Prof. Q. Zhang, Prof. Y. Wang
Beijing Key Laboratory of Molecular Pharmaceutics and New Drug Delivery Systems
School of Pharmaceutical Sciences
Peking University
Beijing 100191, China
Email: yiguang.wang@pku.edu.cn

Y. Yan, Dr. B. Chen, Z. Wang, Q. Yin, Y. Wang, F. Wan, Y. Mo, Dr. B. Xu, Prof. Q. Zhang, Prof. Y. Wang
State Key Laboratory of Natural and Biomimetic Drugs
Peking University
Beijing 100191, China

Experimental section

*Materials***:** The photosensitizer Chlorin e6 (Ce6) was purchased from Frontier Scientific, Inc. (USA). The NHS esters of fluorescent dyes Cy5 and Cy7.5 were obtained from the Lumiprobe Company (Maryland, U.S.A.). The polymers including PEG_{5k}-*b*-poly [2-(ethyl propyl amino) ethyl methacrylate-*r*-2-(propylamino) ethyl methacrylate] (PEG_{5k}-*b*-P(DPA_{40}-*r*-EPA_{40})) and maleimide (MAL)-terminated PEG_{5k}-*b*-P(DPA_{40}-*r*-EPA_{40}) were obtained from our laboratory. N-Hydroxysuccinimide (NHS), Dicyclohexylcarbodiimide (DCC) and 2,2,6,6-Tetramethyl-4-piperidine (TEMP) were purchased from Sigma-Aldrich. Hoechst 33342 was from Invitrogen Inc. (OR, USA). [3-(4, 5-dimethylthiazol-2-yl)-2, 5-diphenyltetrazolium bromide] (MTT) and 2′,7′-dichlorfluorescein-diacetate (DCFH-DA) were supplied by Coolaber (China). N, N-Dimethyl-4-nitrosoaniline (RNO) was supplied by Alfa Aesar (USA). Erbitux was purchased from Merck KgaA (Germany). Anti-Ki67 antibody (ab15580), Fibroblast Marker (ER-TR7) (ab51824), Anti-CD31 antibody [MEC 7.46] (ab7388) and Anti-EGFR antibody [EP38Y] (Alexa Fluor® 488) (ab193244) were purchased from Abcam (Cambridge, MA). Other reagents and solvents were all supported by Sigma-Aldrich or Fisher Scientific Inc.

Cell culture and animals: The human ovarian cancer cell line HO-8910 was purchased from Zhong Qiao Xin Zhou Biotechnology Co.,Ltd. (Shanghai, China), SKOV3 was purchased from the American Type Culture Collection (ATCC), BxPC-3 human pancreatic cancer cell and NIH/3T3 mouse embryonic fibroblast were obtained from National Infrastructure of Cell Line Resource (Beijing, China). These cells were cultured in RPMI 1640 medium (for HO-8910) and DMEM medium (for SKOV3, BxPC-3, NIH/3T3) added with 10% fetal bovine serum (FBS) at 37 °C with 5% CO_2 supply. Female athymic nude mice (18–20 g) were purchased from Vital River Laboratory Animal Center (Beijing, China). The mice were housed under the specific pathogen-free conditions with free access to standard food and water. To establish a HO-8910 tumor model, the suspension of HO-8910 cells (5 ×

10^6 cells/tumor) were injected subcutaneously into the right flanks of the mice. When the tumor volume reached 50 - 100 mm^3, the mice were used for the subsequent *in vivo* fluorescence imaging and photodynamic treatment experiments. All animal procedures were performed according to the Guidelines approved by the Institutional Animal Care and Use Committee (IACUC) of Peking University (Accreditation number: LA2019039).

Syntheses of dye-conjugated polymers: The PEG_{5k}-*b*-P(DPA_{40}-*r*-$PEPA_{40}$-AMA_3) polymer was first synthesized by atom transfer radical polymerization (ATRP) method as described previously.[1] For Ce6 conjugation, Ce6-COOH (5 mg) was first attached with NHS in anhydrous DMF with the presence of coupling reagent DCC to form NHS ester, which was able to react with free amino groups of the polymer (Ce6-COOH: DCC: NHS = 1: 1.1: 1.2, molar ratio). After stirring at room temperature in dark overnight, the polymer (50 mg) was dissolved in 300 μL anhydrous DMF and added to the reaction mixture for another 24 hours. Then the polymer-Ce6 conjugates were purified by preparative gel permeation chromatography (GPC) to remove free dye molecules. The purified polymer conjugates were freeze-dried and kept at -20 °C for storage before further experiments. The similar protocols were applied for the syntheses of Cy5 and Cy7.5-conjugated polymers.

Preparation of the antibody fragment Fab'-SH: Firstly, the pH of Erbitux solution and pepsin enzyme were adjusted to 3.5 with citric acid buffer before mixing. The mixture was digested at 37 °C for 4 h and stopped by neutralizing with 1 mol L^{-1} NaOH.[2] Then, the prepared solution was passed through a Gel filtration column (SUPERDEX 200 10/300 GL) to isolate the $F(ab')_2$ fragments from Fc fragments and other digestion products. The $F(ab')_2$ fragments solution (2 mg) was charged in a reaction bottle, slowly added with 24 μL reductant DTT (DL-Dithiothreitol) and stirred at 37 °C for 4 h under N_2 protection. The obtained solution was applied to a PD10 column and the eluate between 2.5 mL and 5.5 mL was collected to obtain the fresh Fab'-SH fragments.

Preparation and characterization of the antibody-modified micelles: The non-targeted micelles without Fab' modification were prepared according to the previously published procedure.[3] Briefly, PEG_{5k}-*b*-P(DPA_{40}-*r*-$PEPA_{40}$-Ce6) and PEG_{5k}-*b*-P(DPA_{40}-*r*-$PEPA_{40}$-Cy7.5) were mixed in 500 μL THF at a ratio of 1: 1, and then added into 3.5 mL distilled water dropwise under sonication. Finally, the THF was removed through a micro-ultrafiltration system (100 kD) for 4 times to obtain the acid-activatable photosensitizer (AAPS). For the preparation of antibody-modified micelles (Erb-AAPS), a mixture of Mal-PEG_{5k}-*b*-P(DPA_{40}-*r*-EPA_{40}) (0.4 mg), PEG_{5k}-*b*-P(DPA_{40}-*r*-$PEPA_{40}$-Ce6) (3.8 mg) and PEG_{5k}-*b*-P(DPA_{40}-*r*-$PEPA_{40}$-Cy7.5) (3.8 mg) was dissolved in 500 μL THF and then immediately dropped into 3.5 mL Milli-Q deionized water under sonication. Then, THF was removed by ultrafiltration for 4 times to obtain the Mal-AAPS. Subsequently, the freshly prepared Fab'-SH was added into the Mal-AAPS micelle solution at a Fab'-SH: maleimide molar ratio of 2: 1. The resulted mixture was stirred at room temperature overnight in a nitrogen atmosphere. Unconjugated Fab'-SH was removed through 100 kD micro-ultrafiltration system. Finally, Milli-Q deionized water was added to adjust the micelle concentration to 10 mg mL^{-1} as a stock solution and stored at 4 °C before further studies. After micelle formation, transmission electron microscopy (TEM, JEM 1200EX, Japan) was applied to visualize the morphologies of AAPS and Erb-AAPS. The sizes and the zeta potentials of micelles were analyzed by Malvern Zetasizer (Nano ZSP, Malvern, UK).

Acid activatable fluorescence and singlet oxygen generation (SOG): For acid activatable fluorescence investigation, the micelle stock solution was dispersed with citric-phosphate buffers at different pH values (interval 0.2 pH) to Ce6 concentration of 10 μg mL^{-1}, then the fluorescence emission spectra were obtained by a Hitachi fluorescence spectrophotometer (F-7000, Japan). The Ce6 nanoprobes were excited at 400 nm, and corresponding fluorescence emission spectra was collected from 630 to 750 nm. The fluorescence intensity at 670 nm was used to quantify the fluorescence ON/OFF ratio. The UV absorption spectra of AAPS and

Erb-AAPS were determined by a Hitachi UV−Vis spectrometer (UH-5300, Japan). The fluorescence images of micelles with different pH values were captured by an IVIS imaging system (PerkinElmer, USA) using autoexposure with excitation/emission bandpass filter (640 ± 10 nm / 680 ± 20 nm).

The pH-activatable singlet oxygen generation (SOG) was estimated by *p*-nitrosodimethylaniline (RNO) method and electron paramagnetic resonance (EPR) method.[4] For RNO method, imidazole was acted as the $^1O_2$ trapping agent.[5] The AAPS or Erb-AAPS (Ce6 concentration, 10 μg mL^{-1}) was respectively mixed with RNO working solution (50 μM RNO and 10 mM imidazole), followed by irradiation with a 660 nm laser at 100 mW cm^{-2} for 2 min. After irradiation, the RNO consumption at 440 nm was measured as the SOG value. For EPR method, the generated $^1O_2$ could react with 2,2,6,6-tetramethylpiperidine (TEMP), resulting in the paramagnetic 2,2,6,6-tetramethyl-4-piperidone-N-oxyl radical (TEMPO). The AAPS or Erb-AAPS (Ce6 concentration, 10 μg mL^{-1}) was mixed with TEMP working solution (30 mM). Then the micelle solutions were irradiated with a 660 nm laser at 100 mW cm^{-2} for predicted time. Twenty microliter of the micelle samples were taken at each designated time point and transferred into a glass capillary tube for EPR detection with an EPR spectrometer (ESP-300, Bruker, USA).

For stability study, the AAPS or Erb-AAPS stock solution was diluted to 10 μg mL^{-1} (Ce6) with PBS buffer (pH 7.4 and pH 5.4) and fresh mouse plasma in triplicate, respectively. Then, the micelle samples were cultured at 37°C for 24 h, and 200 μL samples were collected at each designated time point. The Ce6 fluorescence intensity and SOG (RNO method) were immediately measured as mentioned above.

Measurement of EGFR expression: Confocal laser scanning microscope (CLSM, Nikon, Japan) and flow cytometry (FCM, Beckman, USA) were applied to evaluate the EGFR expression in various cell lines (HO-8910, SKOV3, BxPC-3 and NIH/3T3). For confocal analysis, cells were seeded in glass bottom dishes and incubated at 37 °C overnight. The cells

were then washed with PBS for three times and fixed with 4% paraformaldehyde for 15 min. Non-specific antibody binding was blocked by pre-incubation with a quick blocking solution at room temperature for 1 h. The cells were then incubated with Alexa Fluor 488-conjugated anti-EGFR antibody at 4 °C overnight. After incubation, cells were washed for three times, followed by nuclei staining with Hoechst 33342. Finally, the fluorescence images were collected by CLSM. For flow cytometry analysis, cells were collected, washed with PBS for three times, blocked with a quick blocking solution and incubated with Alexa Fluor 488-EGFR antibody at 37 °C for 1 h. Subsequently, the cells were washed with PBS to remove the unbound antibodies, followed by detecting with flow cytometry to quantify cell surface EGFR expression. A polyclonal rabbit IgG was used as the isotype control antibody.

In vitro cellular uptake and targeting ability of Erb-AAPS: The cellular uptake of AAPS and Erb-AAPS in HO-8910 cells ($EGFR^{+}$) and NIH/3T3 cells ($EGFR^{-}$) were assessed using CLSM and FCM. Cells were seeded in glass bottom dishes and incubated overnight for attachment. Subsequently, the cells were washed with PBS and incubated with fresh medium (with 10% FBS) containing the AAPS or Erb-AAPS (Ce6 concentration, 10 μg mL^{-1}), respectively. For competitive test, the cells were pre-incubated with 10 μg mL^{-1} Erbitux for 1 h to block the EGFR, followed by incubation with micelles in the presence of excessive Erbitux that acted as a competitive inhibitor of EGFR. After incubation with micelles at 37 °C for 4 h, the cells were washed three times with PBS buffer, and stained with Hoechst 33342 for CLSM analysis. For quantitative analysis of cellular uptake by FCM, cells were seeded in 12-well plates at a density of 2×10^5 cells per well and incubated overnight, followed by incubation with AAPS or Erb-AAPS maintained in fresh medium (with 10% FBS) for 4 h at 37 °C, the competitive experiment was also performed as aforementioned. Afterwards, the cells were washed three times with cold PBS and quantified with FCM.

In vitro cellular SOG of Erb-AAPS: HO-8910 cells were seeded in 12-well plates at a density of 2×10^5 cells per well and incubated overnight. Then, the cells were incubated with

AAPS or Erb-AAPS (Ce6 concentration, 10 μg mL^{-1}) in complete medium at 37 °C for 4 h, followed by dichlorodihydrofluorescein diacetate (DCFH-DA) staining at 37 °C for 20 min. Afterwards, the intracellular singlet oxygen generation (SOG) with/without 660 nm irradiation (100 mW cm^{-2} for 3 min) was assessed by flow cytometry.

In vitro cytotoxicity of Erb-AAPS: For dark toxicity, HO-8910 cells were plated in 96-well plates at a density of 2×10^4 cells per well and incubated overnight. The supernatant was then replaced with fresh RPMI 1640 medium (with 10% FBS) containing series concentrations of Erb-AAPS. After incubation at 37 °C in the dark for 24 h, the cell viability was evaluated by MTT assay. Briefly, the supernatant was aspirated and displaced with 150 μL MTT working solution (0.5 mg mL^{-1}). After 4 h incubation at 37 °C, the MTT solution was discarded and 150 μL DMSO was added to dissolve the generated formazan. Finally, the OD values at 560 nm were measured with a microplate reader to evaluate the cytotoxicity.

For *in vitro* photodynamic therapy (PDT) evaluation, HO-8910 cells and NIH/3T3 cells were plated in 24-well plates at a density of 1×10^5 cells/well and incubated overnight. Then, the supernatant was replaced with fresh complete culture medium supplemented with series concentrations of AAPS or Erb-AAPS. After 4 h incubation, the micelle containing medium was discarded and replaced with fresh complete culture medium without phenol red. Afterwards, the cells were irradiated with a 660 nm laser at 100 mW cm^{-2} for 3 min. The PDT effect was assessed by MTT assay at 24 h post irradiation. The IC_{50} values of micelles were calculated by Origin software. In addition, the PDT induced cell apoptosis was evaluated with Annexin V-FITC/PI Apoptosis Detection Kit according to the manufacturer's instructions, and respectively analyzed by CLSM and FCM, respectively.

Tumor targeting behavior analyzed by in vivo fluorescence imaging: For *in vivo* imaging, the Cy5 probe with superior fluorescence properties was used as a substitute of Ce6 to monitor the AAPS and Erb-AAPS micelles. The *in vivo* tumor targeting capability of AAPS and Erb-AAPS micelles were evaluated in an ovarian cancer tumor mode which was

established as described above. HO-8910 tumor bearing mice were injected intravenously with micelles at an equivalent Cy5 concentration of 3 mg kg^{-1}. At 3, 6, 12 and 24 h post-injection, the fluorescence images of mice were captured by an *in vivo* imaging system (PerkinElmer, USA) using autoexposure with excitation/emission bandpass filter (640 ± 10 nm / 680 ± 20 nm). For thalidomide (THD) regulation study, the mice of THD regulation groups were orally treated with a low dose of THD (50 mg kg^{-1}) for three consecutive days before micelles administration. THD was dispersed in PBS containing 0.1% (w/v) SDS. The mice of other groups were orally treated with PBS (containing 0.1% SDS) as control.

For pre-PDT regulation study, a bilateral tumor model was established by injecting HO-8910 cells to the bilateral flanks of the mouse. After the tumor volume reached around 100 mm^3, mice were administrated with AAPS micelles (Ce6 concentration of 3 mg kg^{-1}) intravenously for pre-PDT treatment. At 3 h post-injection, the tumors implanted on the right flanks were irradiated with a 660 nm laser for 10 minutes (400 mM cm^{-2}). The tumors implanted on the left flanks were kept in dark as control. Then, 36 h after PDT treatment, the mice were injected intravenously with imaging micelles (Cy5 concentration of 3 mg kg^{-1}), and imaged at different time post-injection by an *in vivo* imaging system. At 24 h post-injection, the mice were sacrificed, and the organs were excised for *ex vivo* fluorescence imaging. After imaging, the excised tumors were further sliced or dissociated for immunochemistry and FCM study, respectively.

In vivo targeting behavior analyzed by immunochemistry: The tumor slices in above *in vivo* imaging section were fixed in 4% paraformaldehyde, permeabilized with 0.1% Triton X-100 for 5 min and then blocked with quick blocking solution for 1 h at room temperature. Afterwards, the slices were incubated with rat anti-ER-TR7 antibody (fibroblast marker) at 4 °C overnight. Then PE-conjugated secondary antibody and Alexa Flour 488-conjugated rabbit anti-EGFR primary antibody were added together to stain the tumor slices at 37 °C for 2 h. In addition, an adjacent slice was incubated with rat anti-CD31 primary antibody,

followed by Alexa Flour 488-conjugated secondary antibody. Finally, the slides were stained with Hoechst 33342 and scanned by a quantitative slide scanner (PerkinElmer Vectra Polaris).

In vivo targeting behavior analyzed by FCM: The excised tumors in above *in vivo* imaging section were cut into small pieces, followed by digestion with 0.5 mg mL^{-1} collagenase I and 0.2 mg mL^{-1} DNAase I in sterile serum-free RAMI 1640 media at 37 °C for 30 min. Then the suspension was filtered through a 100-μm nylon mesh to obtain the single-cell suspension. After centrifugation at 300 g for 5 min, the supernatant was removed and red blood cell (RBC) lysis buffer was added to lyse red blood cells on ice for 2 min. Subsequently, the cells were washed with cell staining buffer for three times, followed by incubation with anti-CD16/32 to block the nonspecific binding to FcRs. Afterwards, the cells were incubated with rat anti-ER-TR7-PE and rabbit anti-EGFR-AF488 antibodies at 37 °C for 0.5 h. Finally, the cells were washed thrice with cell staining buffer and resuspended in 400 μL cell staining buffer for FCM analysis.

Cellular uptake of Erb-AAPS by CAFs under co-cultured with HO-8910 tumor cells: NIH/3T3 fibroblasts were cocultured with HO-8910 tumor cells at 37 °C to simulate the cancer associated fibroblasts (CAFs) *in vitro*. After 48 h incubation, the culture medium was replaced with fresh medium (with 10% FBS) containing the Erb-AAPS micelles for another 4 h. Then, the co-cultured cells were washed three times with PBS, followed by 4% paraformaldehyde fixation. Subsequently, the CAFs and tumor cells were stained by rat anti-ER-TR7-PE and rabbit anti-EGFR-AF488 antibodies at 4 °C overnight, respectively. Finally, the cellular uptake of Erb-AAPS micelles by HO-8910 cells and CAFs was observed by CLSM. The immunofluorescence experiment was also performed for mono-HO-8910 cells and mono-NIH/3T3 normal fibroblasts (NFs) as control.

Tumor blood flow study: The tumor blood flow status was investigated by Laser Doppler imaging system (moorLDLS2, UK) after the HO-8910 tumor-bearing mice were treated with THD (50 mg kg^{-1}) via oral gavage for three consecutive days.[6] The mice of different groups

were anesthetized with avertin before the measurement. The distance between the surface of the mouse's body and the scanner was adjust to 14 cm, each mouse was recorded for three times and averaged to calculate the blood flow in tumor. The final images and results were analyzed using mLDLSMainV21 software.

In vivo anti-tumor efficacy of Erb-AAPS: HO-8910 tumor-bearing nude mice were randomly divided into four groups (n = 6 for each group), and treated with PBS, Erb-AAPS, AAPS+IR and Erb-AAPS+IR, respectively (IR: laser irradiation). The PBS or micelles (Ce6 dose of 3 mg kg^{-1}) were administrated via tail vein injection. For laser irradiated groups, mice were anesthetized at 3 h post-injection of AAPS or Erb-AAPS, then the tumors of mice were performed a single irradiation with 660 nm laser at a laser power of 400 mW cm^{-2} for 10 min. The tumor sizes were measured every other day using a digital caliper, and the body weight change of each mouse was monitored over a time course of 27 d. At 36 h after PDT treatment, one mouse of each group was euthanized and the tumor was harvested, sliced and stained for Ki67 and CAFs, separately.

For thalidomide (THD) regulation study, HO-8910 tumor-bearing nude mice were randomly divided into six groups (n = 5 for each group), and treated with PBS, THD+IR, AAPS+IR, Erb-AAPS+IR, THD+AAPS+IR and THD+Erb-AAPS+IR, respectively. The mice were orally treated with THD (50 mg kg^{-1}) for three consecutive days in THD regulation groups before micelles administration. THD was dispersed in PBS containing 0.1% (w/v) SDS. The mice of AAPS+IR and Erb-AAPS+IR groups were orally treated with PBS (containing 0.1% SDS) as control. The laser irradiation was performed as aforementioned.

For pre-PDT regulation study, HO-8910 tumor-bearing nude mice were randomly divided into five groups (n = 5 for each group), and treated with PBS, AAPS+IR, Erb-AAPS+IR, pre-PDT+AAPS+IR and pre-PDT+Erb-AAPS+IR, respectively. For pre-PDT treated groups, the mice were performed an additional PDT treatment with AAPS at 36 h before subsequent PDT treatment, the mice were injected intravenously with AAPS (Ce6 dose of 3 mg kg^{-1}), followed

by irradiation with a 660 nm laser for 10 minutes (400 mM cm^{-2}) at 3 h post-injection. The subsequent PDT treatment was the same as described above. The tumors were collected, weighted and pictured at the end of anti-tumor study.

For combined THD and pre-PDT regulation study, HO-8910 tumor-bearing nude mice were randomly divided into five groups (n = 7 for each group), and treated with PBS, AAPS+IR, Erb-AAPS+IR, THD+pre-PDT+AAPS+IR and THD+pre-PDT+Erb-AAPS+IR, respectively. For THD+pre-PDT+AAPS+IR and THD+pre-PDT+Erb-AAPS+IR groups, the mice were orally treated with THD (50 mg kg^{-1}) for three consecutive days on day 0, 1 and 2, followed by pre-PDT treatment on day 3. The subsequent PDT treatment was performed on day 5 which was the same as described above. The survival curve of each group was recorded during the antitumor study. At 2 days after the last PDT treatment, one mouse of each group was euthanized and the tumors were harvested and sliced for TUNEL and hematoxylin-eosin (H&E) staining, separately. The major organs of mice were excised for histological study by H&E staining to evaluate the systemic toxicity at day 30. In addition, whole blood samples were collected at 24 h post the last PDT treatment for routine blood analysis.

Statistical analysis: Data were presented as mean ± s.d. All experiments were performed in triplicate at least. Statistical significance was determined using an unpaired student's t-test or two-way analysis of variance (ANOVA) with Tukey’s post-hoc test in GraphPad Prism 7 software. The comparison of survival curves was analyzed using a log-rank test. In all cases, statistical significance was set as follows: *$p<0.05$ was considered significant, **$p<0.01$ was regarded as highly significant.

Supporting Table and Figures

Table S1. Characterization of dye-conjugated polymeric micelles

UPS-Dye$_x$	R_F (F_{max}/F_{min})	pH_t	△$pH_{10\text{-}90\%}$	Conjugation Number
UPS-Ce6$_3$	4.02	6.48	0.19	2.1
UPS-Cy7.5$_3$	68.6	6.42	0.20	2.7
UPS-Cy5$_3$	83.25	6.47	0.17	2.4

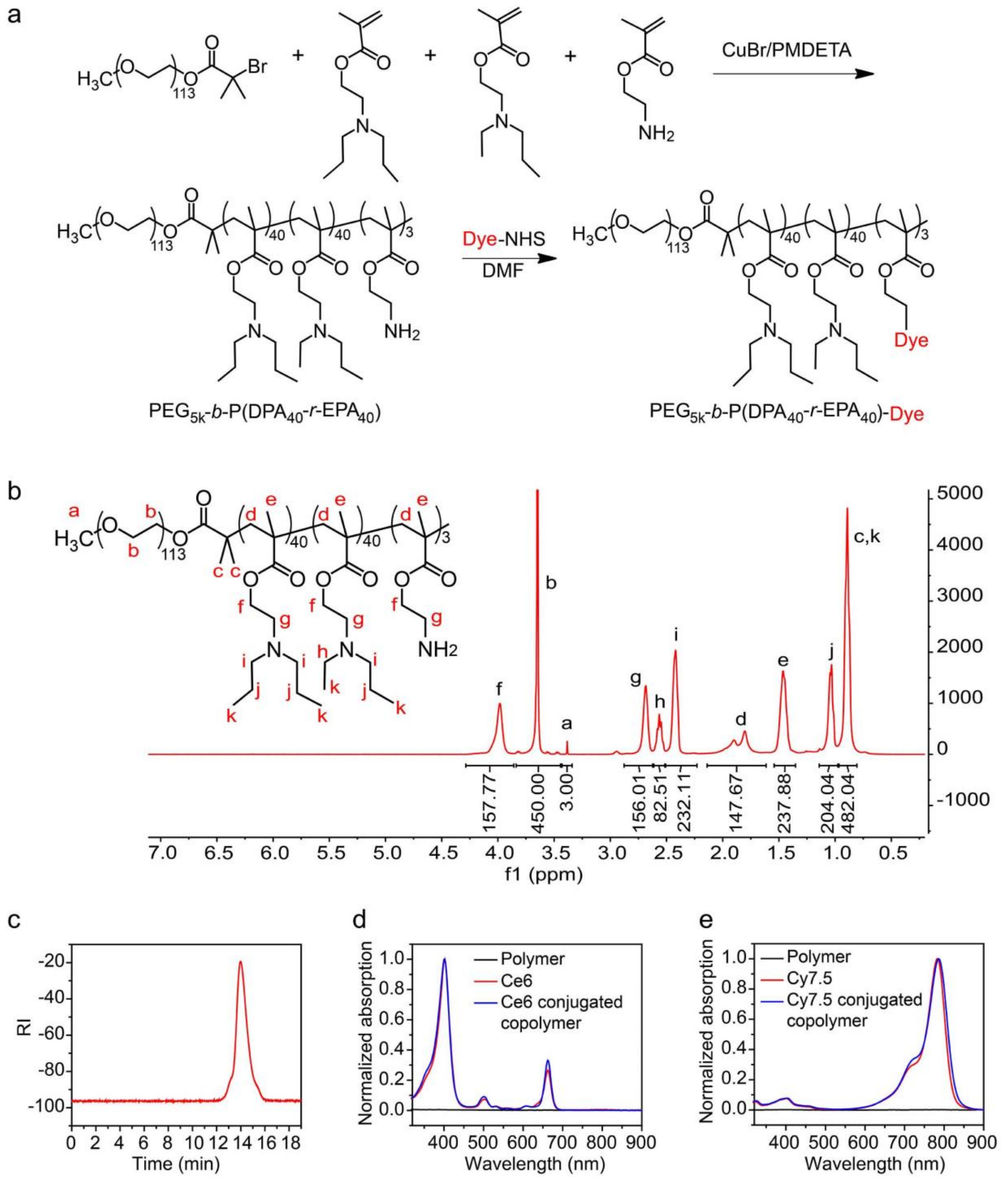

Figure S1. The characterization of PEG$_{5k}$-*b*-P(DPA$_{40}$-*r*-EPA$_{40}$) and dye-conjugated copolymers. (a) Synthetic routes of PEG$_{5k}$-*b*-P(DPA$_{40}$-*r*-EPA$_{40}$) and dye-conjugated block copolymers. (b-c) ^{1}H-NMR spectrum (b) and GPC profile (c) of PEG$_{5k}$-*b*-P(DPA$_{40}$-*r*-EPA$_{40}$). The molecular weight of the copolymer was 21.5 kDa, calculating from the ^{1}H-NMR. (d-e) Normalized UV-Vis spectra of free dyes, copolymers and dye-conjugated copolymers.

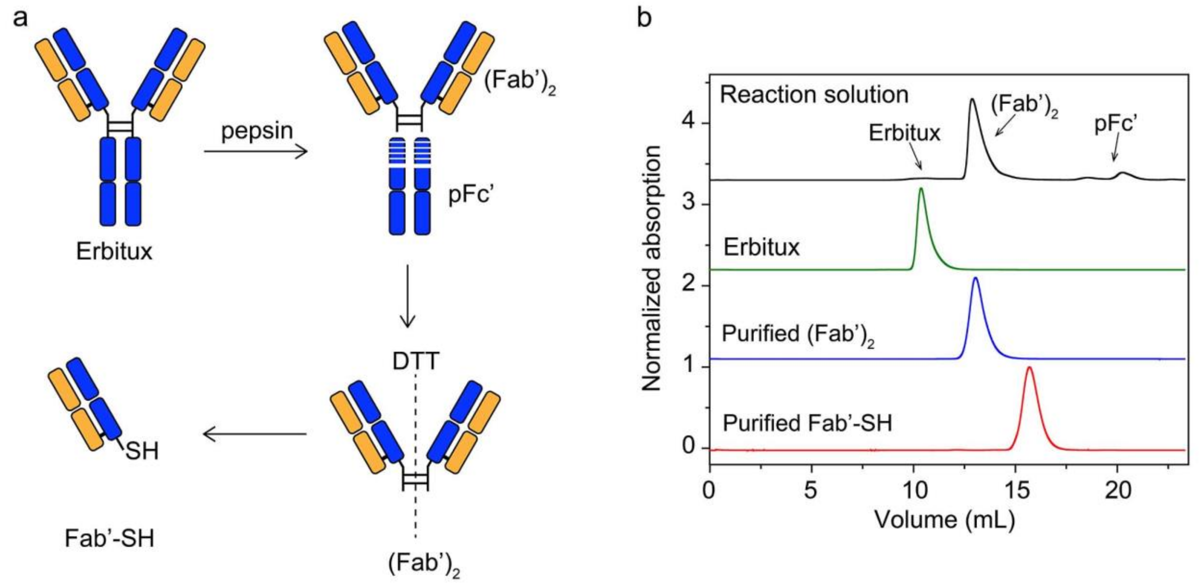

Figure S2. The preparation of Fab'-SH fragments. (a) Schematic illustration of the preparation steps of Fab' fragments. (b) The Fast Protein Liquid Chromatography (FPLC) profiles of reaction solution, Erbitux, purified $(Fab')_2$ and purified Fab'-SH.

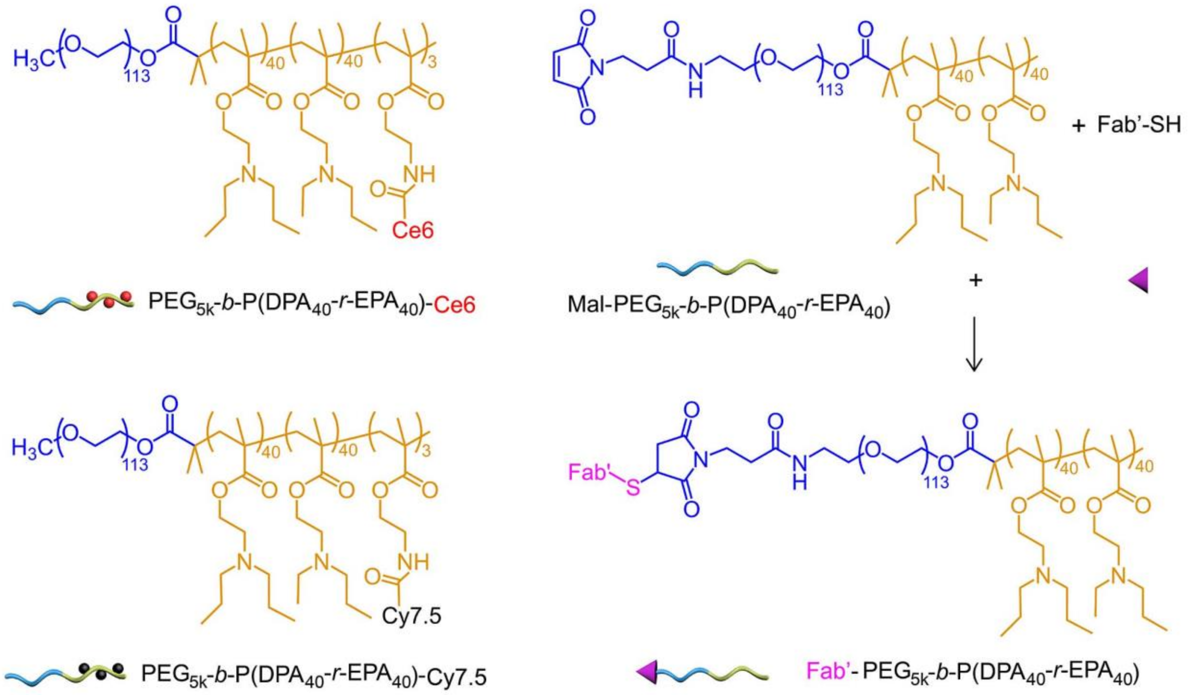

Figure S3. The chemical structures of functionalized block copolymers, including PEG$_{5k}$-*b*-P(DPA$_{40}$-*r*-EPA$_{40}$)-Ce6, maleimide-terminated PEG$_{5k}$-*b*-P(DPA$_{40}$-*r*-EPA$_{40}$), Fab'-conjugated PEG$_{5k}$-*b*-P(DPA$_{40}$-*r*-EPA$_{40}$), and PEG$_{5k}$-*b*-P(DPA$_{40}$-*r*-EPA$_{40}$)-Cy7.5.

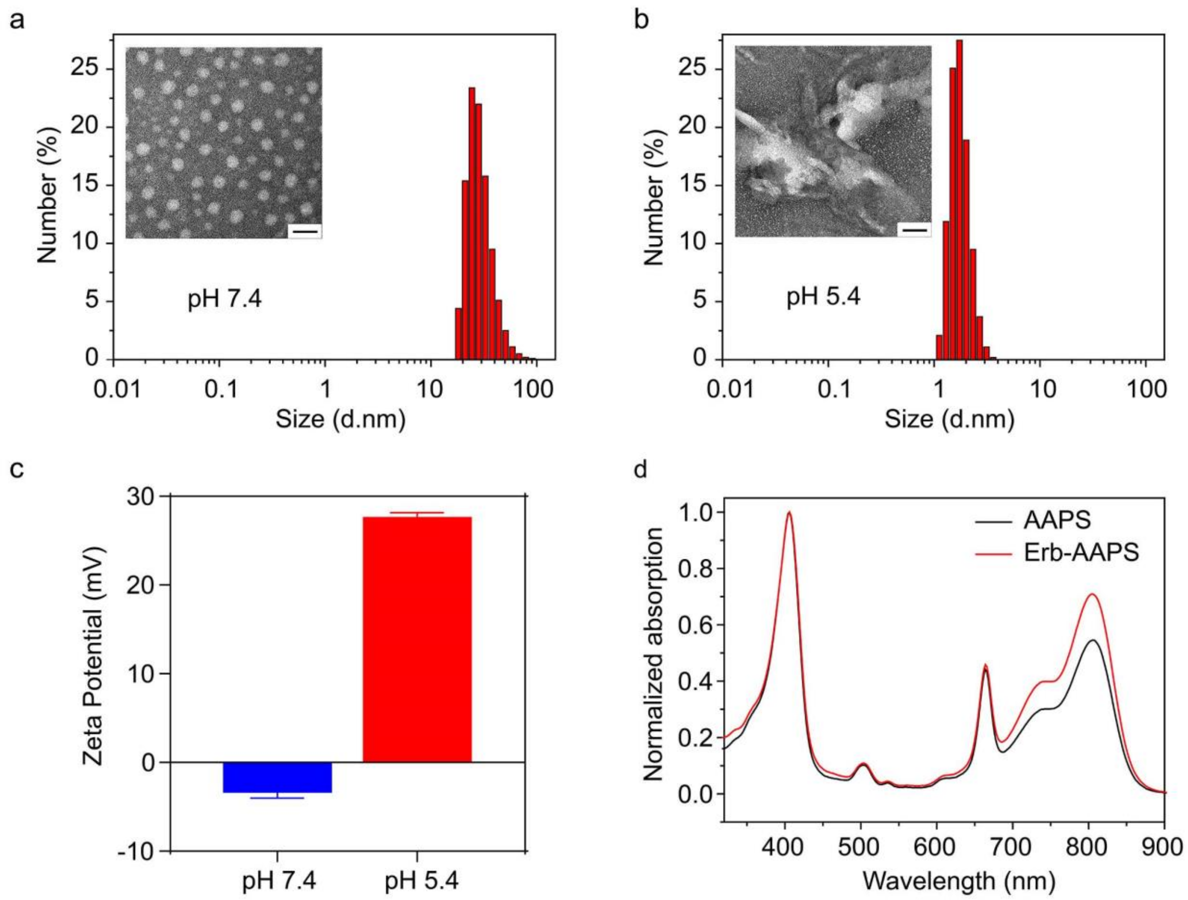

Figure S4. Characterization of AAPS. (a-b) The particle size distribution and TEM images of AAPS at pH 7.4 (a) and pH 5.4 (b), respectively. Scale bar = 50 nm. (c) Zeta potentials of AAPS at pH 7.4 and pH 5.4. Data were presented as mean ± s.d. (n = 3). (d) Normalized UV spectra of AAPS and Erb-AAPS at pH 7.4.

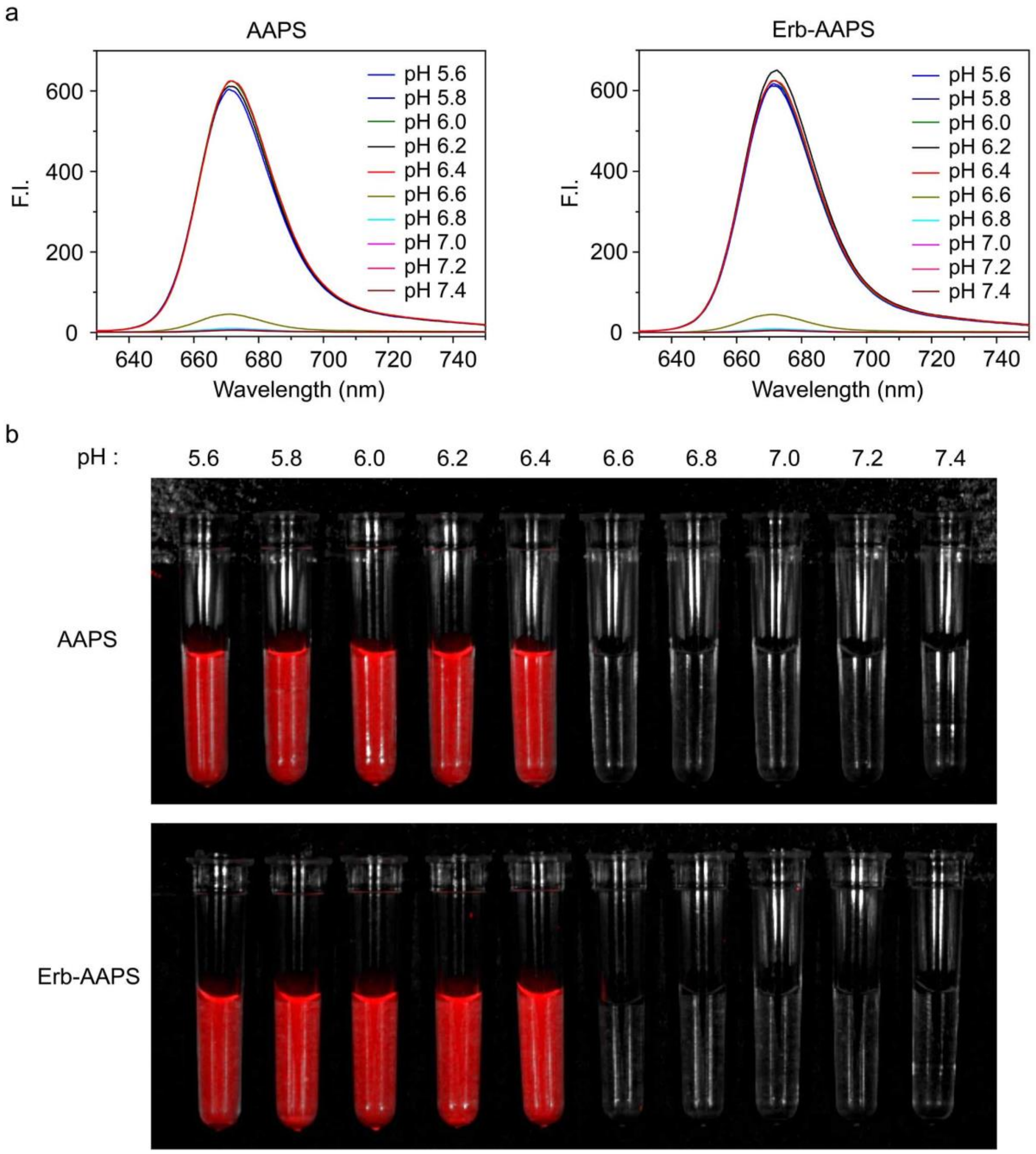

Figure S5. pH-triggered Ce6 fluorescence activation of AAPS and Erb-AAPS. (a) pH-dependent fluorescence spectra of AAPS and Erb-AAPS. The fluorescence intensity of Ce6 was measured within a pH range of 5.6 - 7.4. Each sample was excited at 400 nm, and the corresponding emission spectrum was collected from 630 to 750 nm. (b) Fluorescence images of AAPS and Erb-AAPS at different pH values, recorded by an IVIS system (λ_{ex} = 640 nm and λ_{em} = 680 nm).

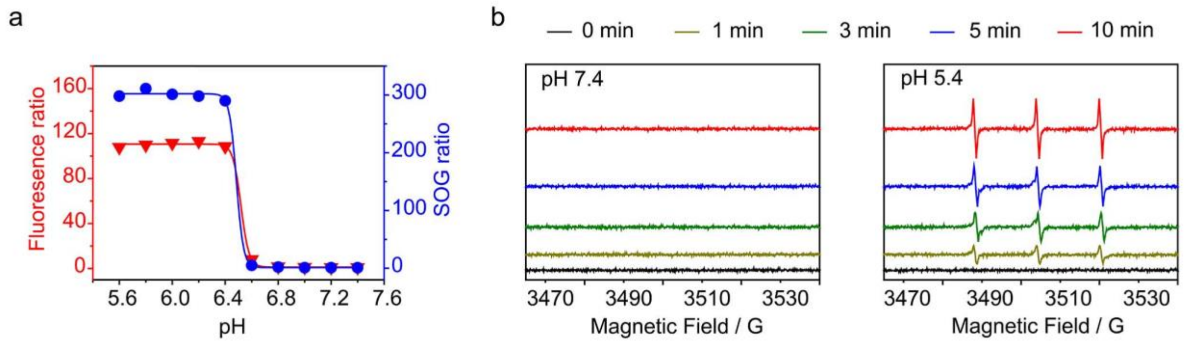

Figure S6. The pH-triggered SOG activation of AAPS. (a) The Ce6 fluorescence ratio and SOG ratio (F_{pH} / $F_{pH\ 7.4}$) of AAPS as a function of pH (Ce6 concentration, 10 μg mL^{-1}). The SOG was estimated by *p*-nitrosodimethylaniline (RNO) method with 660 nm irradiation at 100 mW cm^{-2} for 2 min. (b) The SOG of AAPS detected by EPR spectroscopy when irradiated with 660 nm laser at 100 mW cm^{-2} for different time.

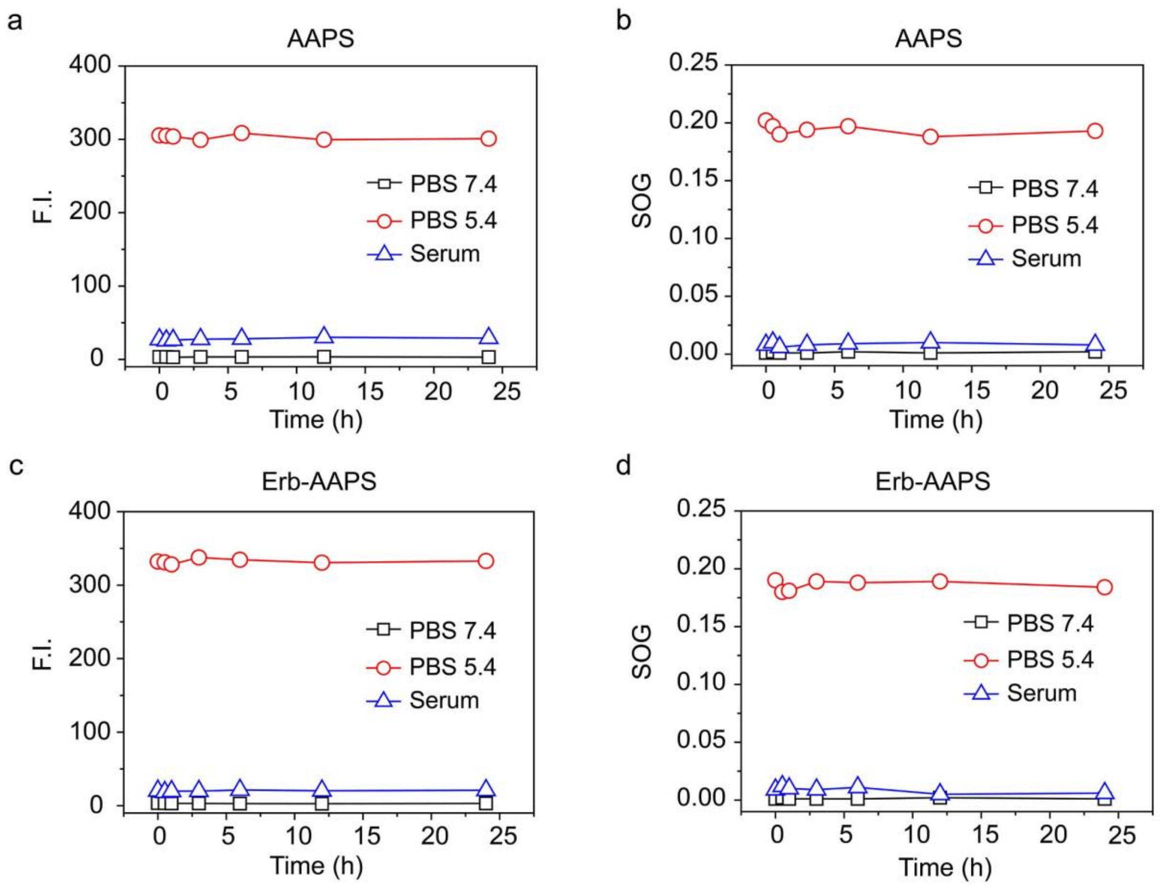

Figure S7. The stability of AAPS and Erb-AAPS. (a-b) The Ce6 fluorescence intensity (a) and SOG (b) of AAPS (Ce6 concentration, 10 μg mL^{-1}) in PBS 5.4 buffer, PBS 7.4 buffer and plasma over a time course of 24 h, respectively. (c-d) The Ce6 fluorescence intensity (c) and SOG (d) of Erb-AAPS in PBS 5.4 buffer, PBS 7.4 buffer and plasma over a time course of 24 h, respectively. The fluorescence emission at 670 nm was used as the value of Ce6 fluorescence intensity, and the RNO consumption at 440 nm was measured as the SOG value.

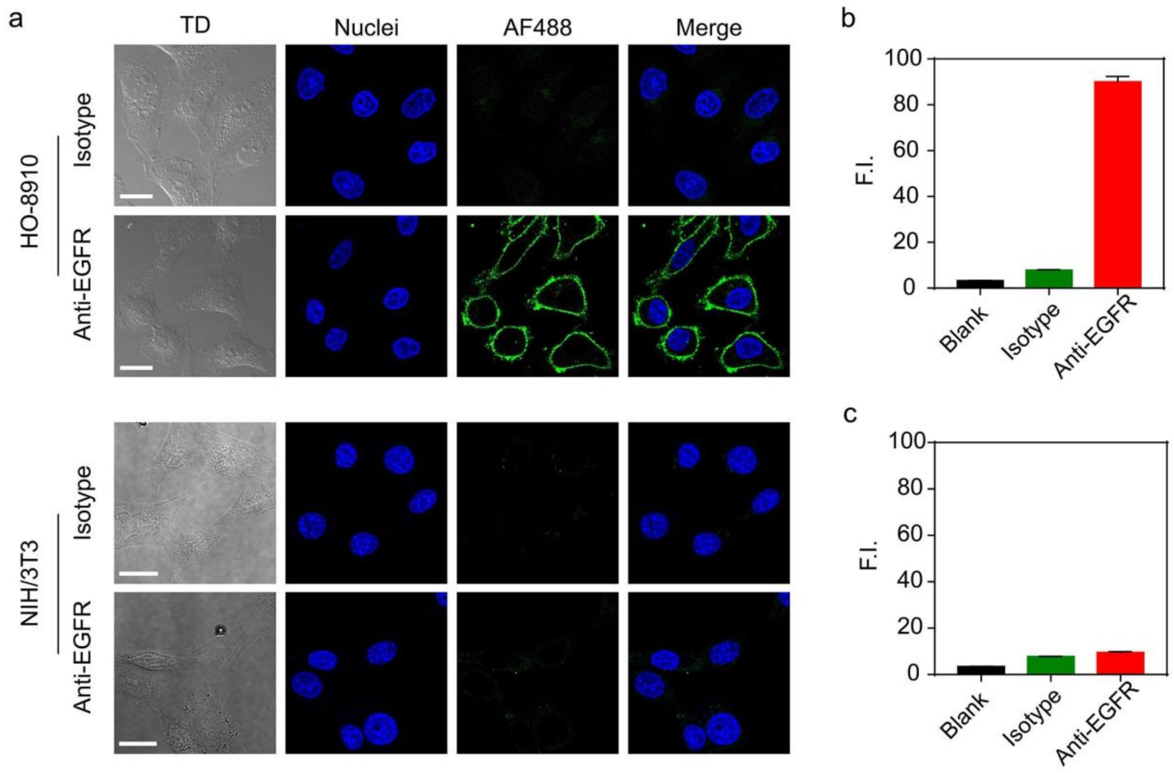

Figure S8. The expression of EGFR on HO-8910 cells and NIH/3T3 cells. (a) Immunohistochemical staining of EGFR expression on HO-8910 ovarian cancer cells and NIH/3T3 fibroblasts. The EGFR and cell nuclei were stained with Alexa Fluor 488-EGFR antibody and Hoechst 33342, respectively. A polyclonal rabbit IgG was used as the isotype control antibody. Scale bars = 20 μm. (b-c) Quantitative EGFR expression on HO-8910 ovarian cancer cells (b) and NIH/3T3 fibroblasts by flow cytometry (c). Data were presented as mean ± s.d. (n = 3).

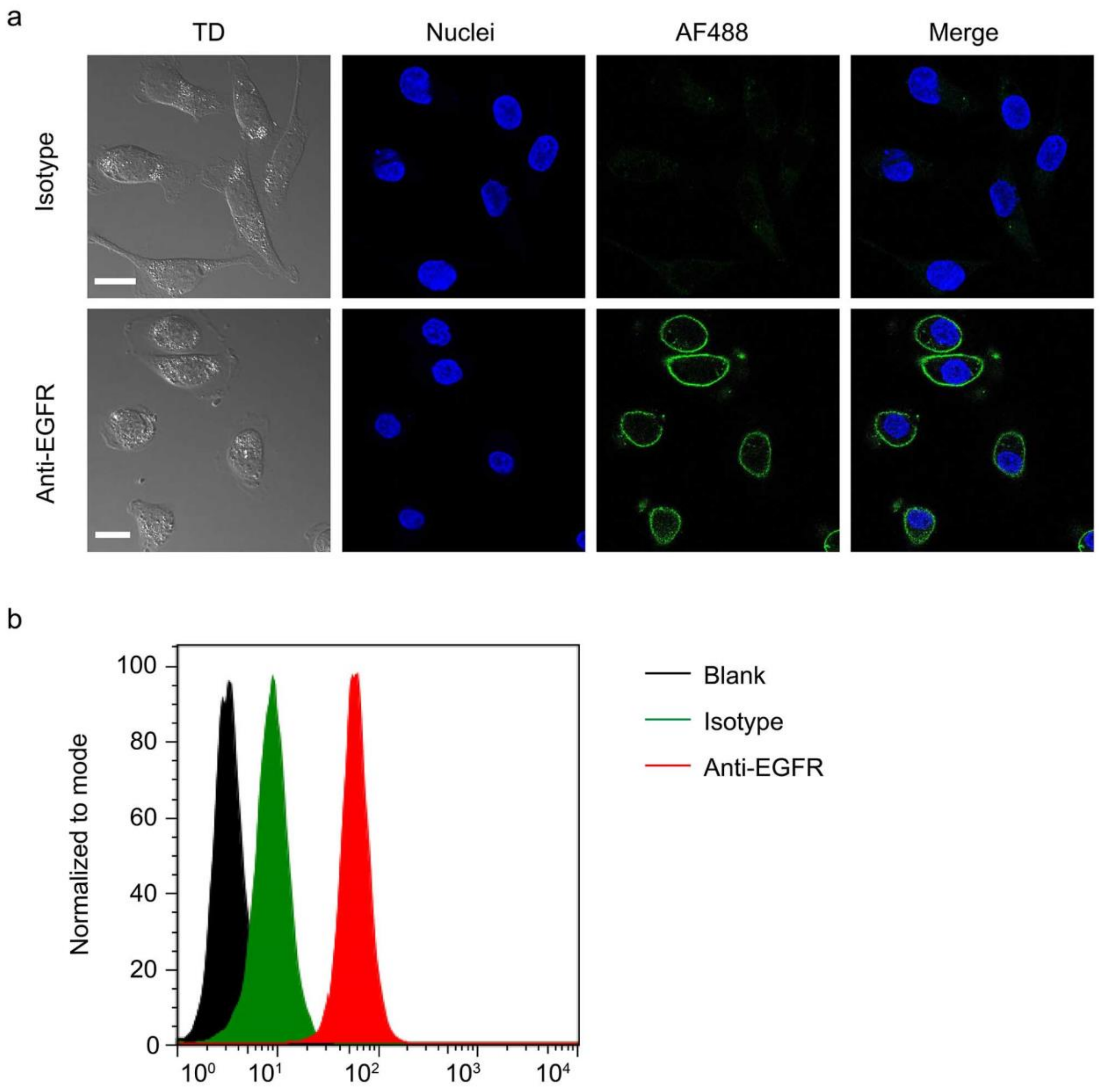

Figure S9. The EGFR expression of SKOV3 cells. (a) Immunohistochemical staining of EGFR expression on SKOV3 ovarian cancer cells. The EGFR and cell nuclei were stained with Alexa Fluor 488-EGFR antibody and Hoechst 33342, respectively. A polyclonal rabbit IgG was used as the isotype control antibody. Scale bar = 20 μm. (b) EGFR expression of SKOV3 ovarian cancer cells quantified by flow cytometry.

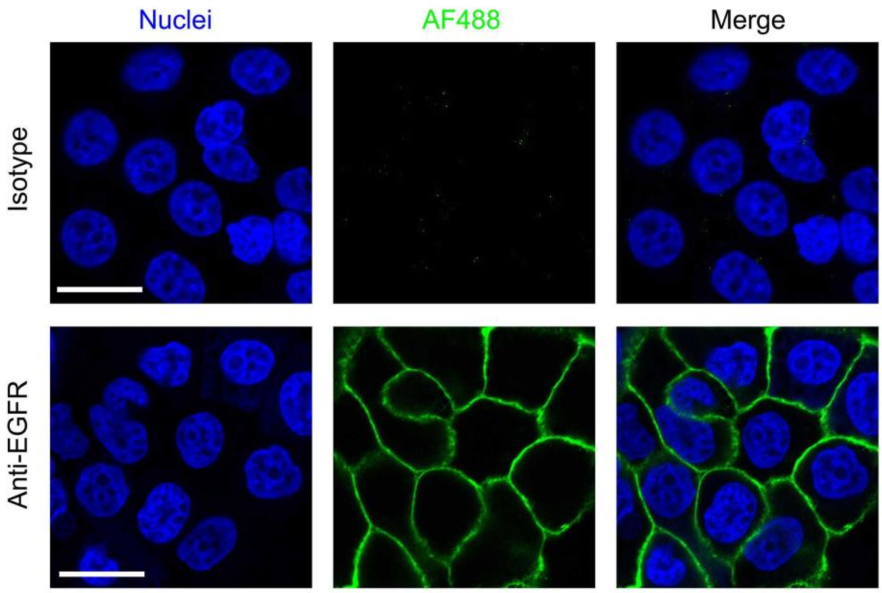

Figure S10. The EGFR expression of BxPC-3 cells. Immunohistochemical staining of EGFR expression on BxPC-3 cells. The EGFR and cell nuclei were stained with Alexa Fluor 488-EGFR antibody and Hoechst 33342, respectively. A polyclonal rabbit IgG was used as the isotype control antibody. Scale bar = 20 μm.

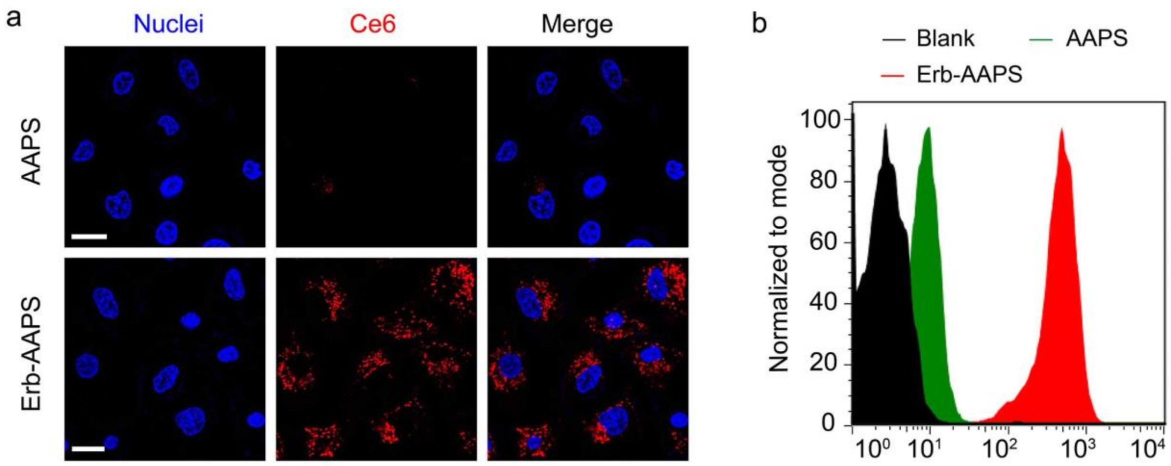

Figure S11. The cellular uptake of AAPS and Erb-AAPS in EGFR-positive SKOV3 cells. (a) The confocal images of the cellular uptake of AAPS and Erb-AAPS (Ce6 concentration, 10 μg mL^{-1}) after incubation with EGFR-positive SKOV3 cells at 37 °C for 4 h. Scale bar = 20 μm. (b) Quantitative cellular uptake of AAPS and Erb-AAPS in SKOV3 cells analyzed by flow cytometry.

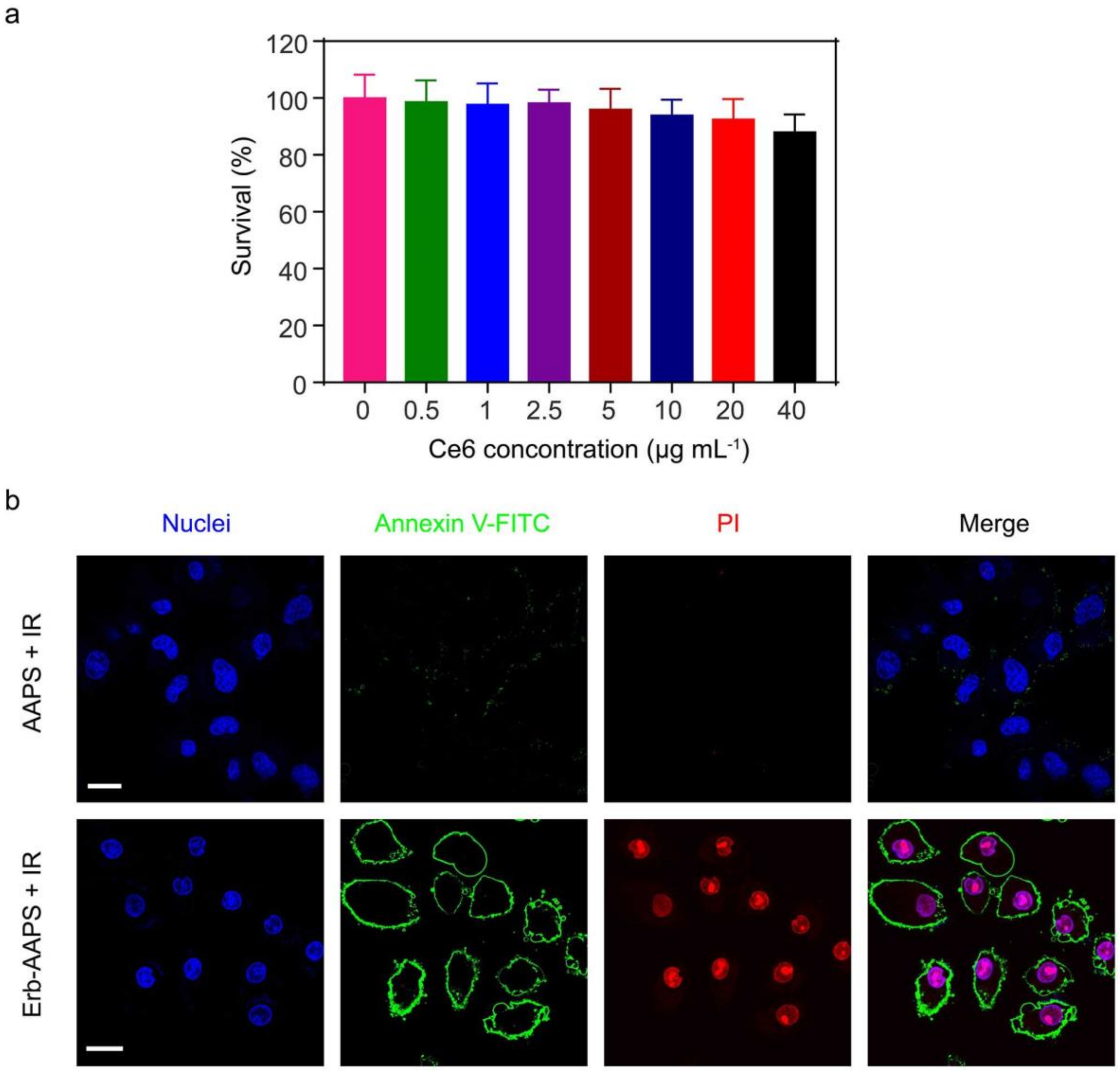

Figure S12. *In vitro* cytotoxicity of AAPS and Erb-AAPS. (a) The dark toxicity of Erb-AAPS measured by MTT assay. HO-8910 cells were incubated with fresh RPMI 1640 medium (with 10% FBS) containing series concentration of Erb-AAPS. After incubation at 37 °C in the dark for 24 h, the cell viability was evaluated by MTT assay. Data were presented as mean ± s.d. (*n* = 3). (b) Confocal images of SKOV3 cell apoptosis induced by AAPS or Erb-AAPS-mediated PDT indicated with Annexin V and PI staining. Scale bar = 20 μm.

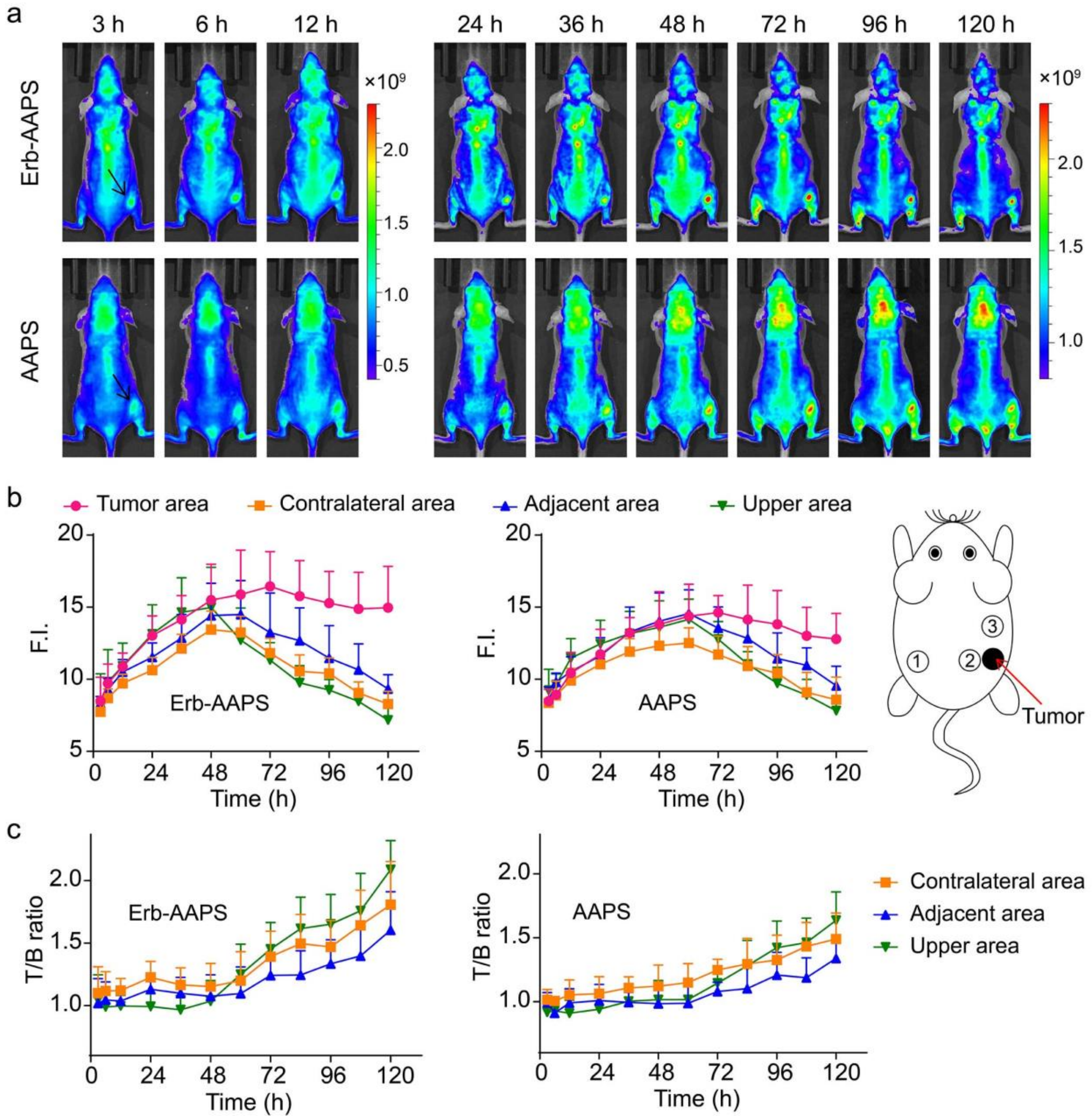

Figure S13. (a) *In vivo* fluorescence images of the HO-8910 tumor-bearing mice at different time post-injection of AAPS and Erb-AAPS micelles. The tumor regions were indicated by the black arrows. (b) The quantitative results of signal measured in tumor and three other locations (the contralateral area, adjacent area and upper area of the tumor) of the body over time, the three areas were marked with circles in the body of the mouse in the right panel. Data are presented as mean ± s.d. (n = 4). (c) The fluorescence intensity ratio between tumor and other body sites (T/B ratio) as a function of time after AAPS or Erb-AAPS injection. Data are presented as mean ± s.d. (n = 4).

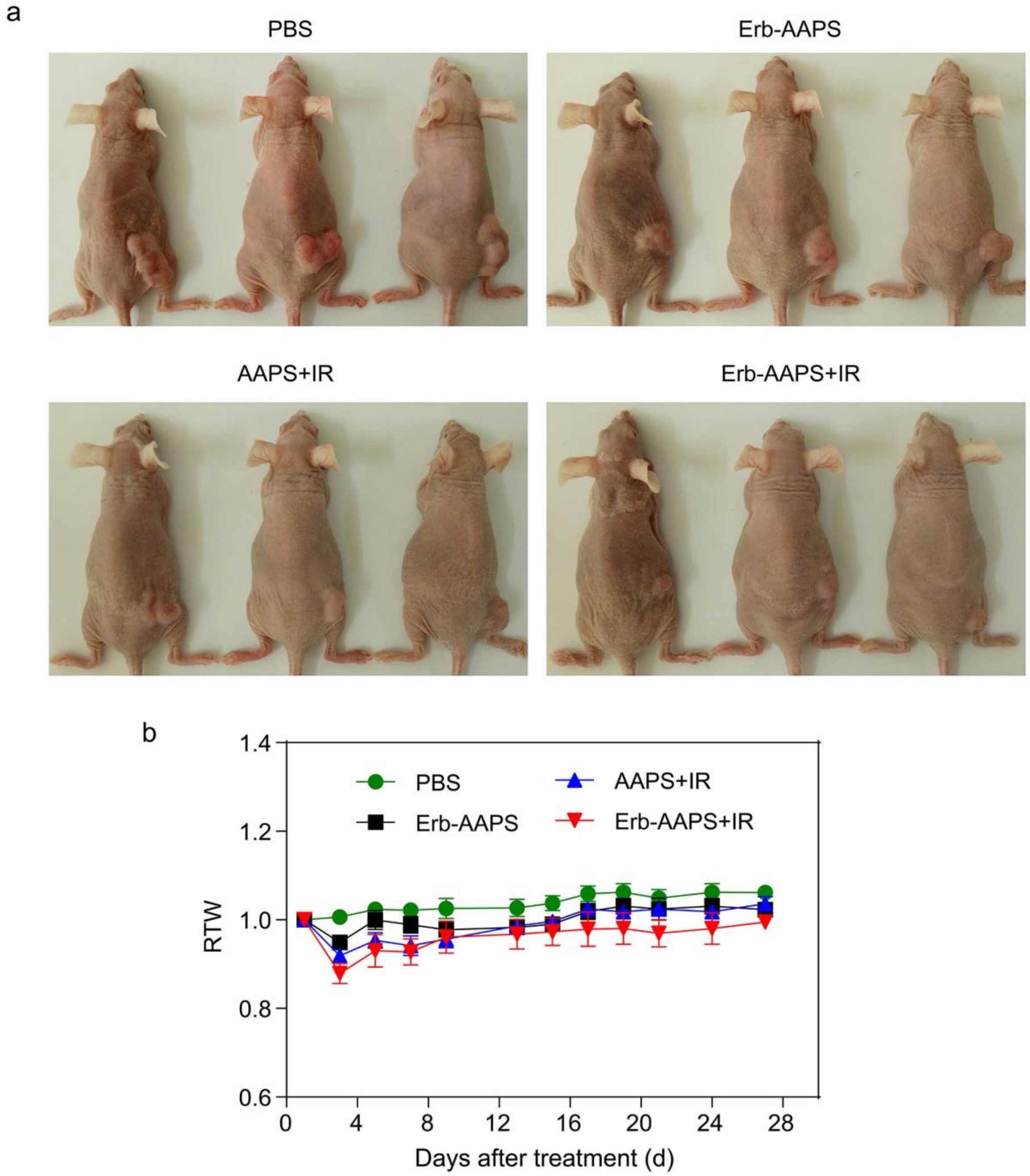

Figure S14. (a) The photographs of tumors in different treatment groups at the end of anti-tumor study. (b) The body weight changes of mice during the anti-tumor study. Data were presented as mean ± s.d. (n = 5).

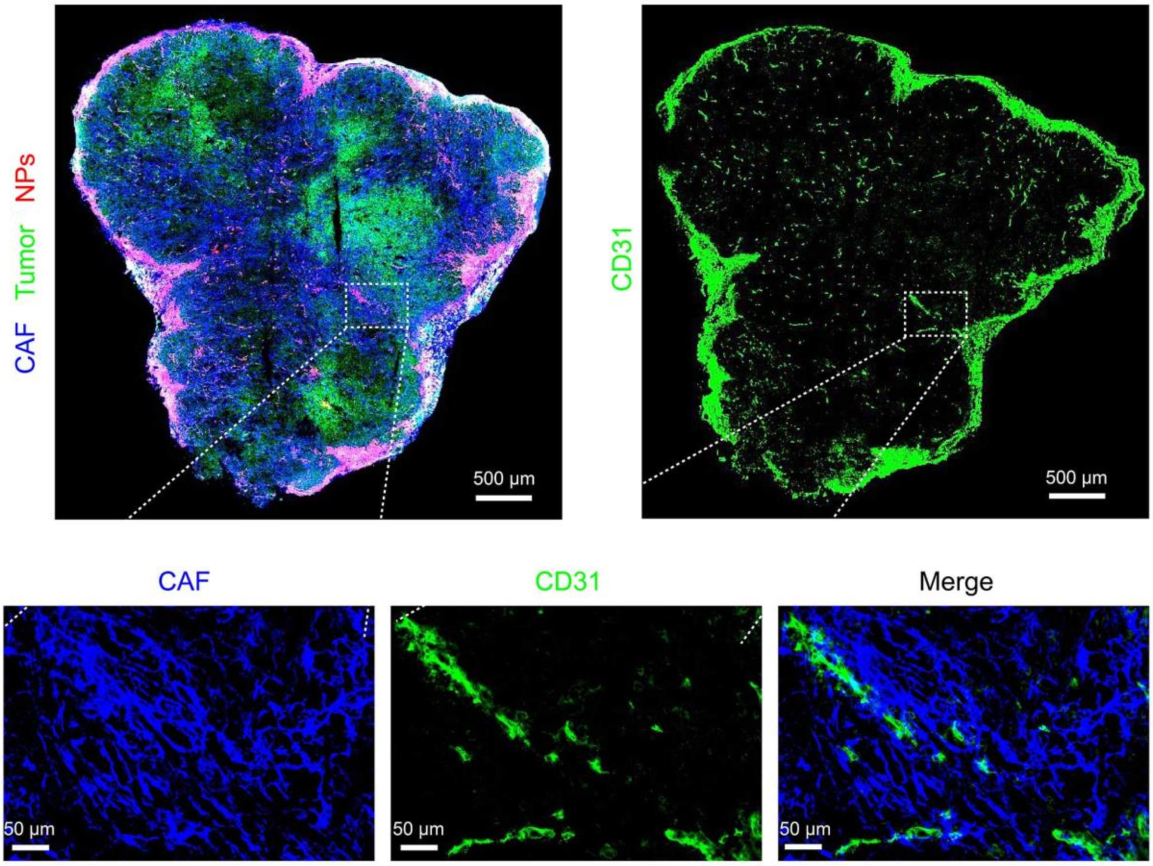

Figure S15. The stroma-vessel architecture of HO-8910 tumor. The HO-8910 tumor slice was stained with anti-ER-TR7 antibody (fibroblast marker) and anti-EGFR antibody (HO-8910 tumor cell marker) at 24 h post injection of Erb-AAPS nanoparticles (NPs). In addition, an adjacent slice was incubated with anti-CD31 antibody for tumor blood vessels staining. CAF, HO-8910 and NPs were denoted as blue, green, and red signals, respectively. For the adjacent slice, the tumor blood vessels were presented in green.

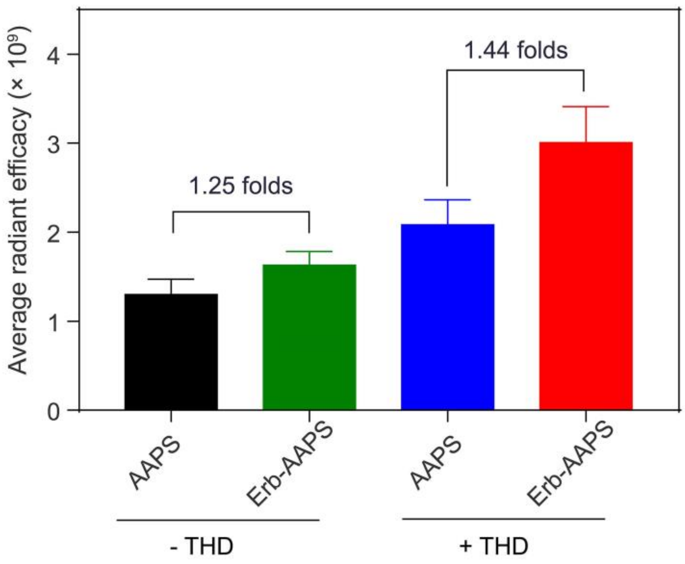

Figure S16. The quantitative intratumoral accumulation of micelles in HO-8910 tumors with or without THD treatments. Data were presented as mean ± s.d. (n = 3).

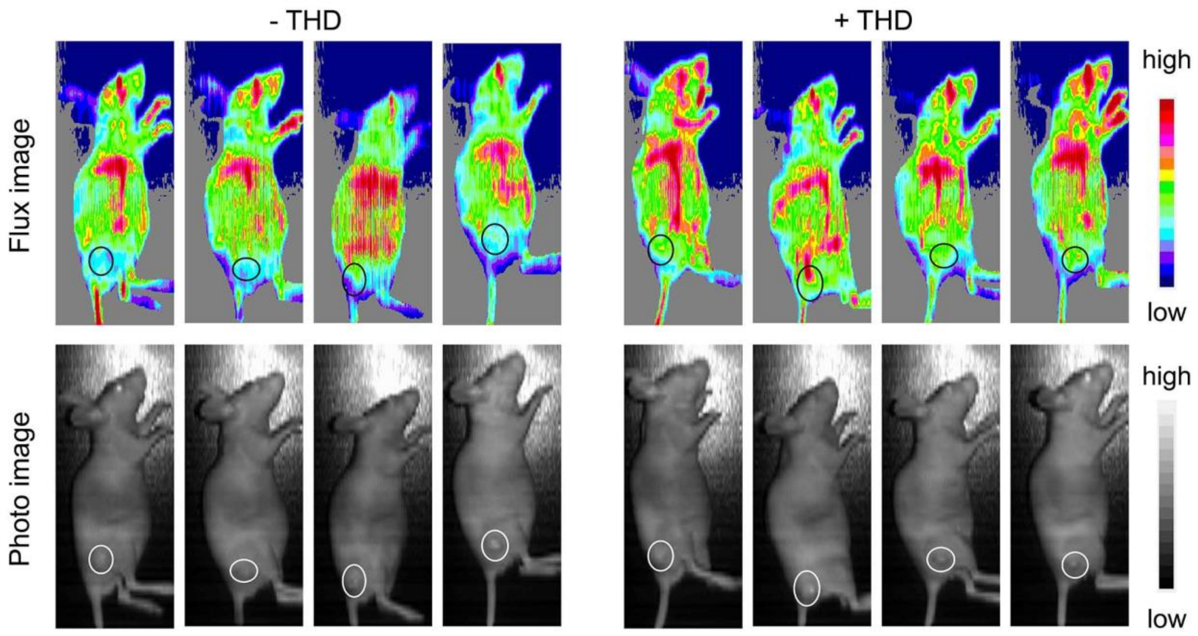

Figure S17. Tumor blood flow of HO-8910 tumor-bearing mice with or without THD treatments, measured by Laser Doppler imaging system. Mice were treated with THD (50 mg kg^{-1}) for three consecutive days before blood flux measurement. Tumor regions were marked by circles in flux images and photo images.

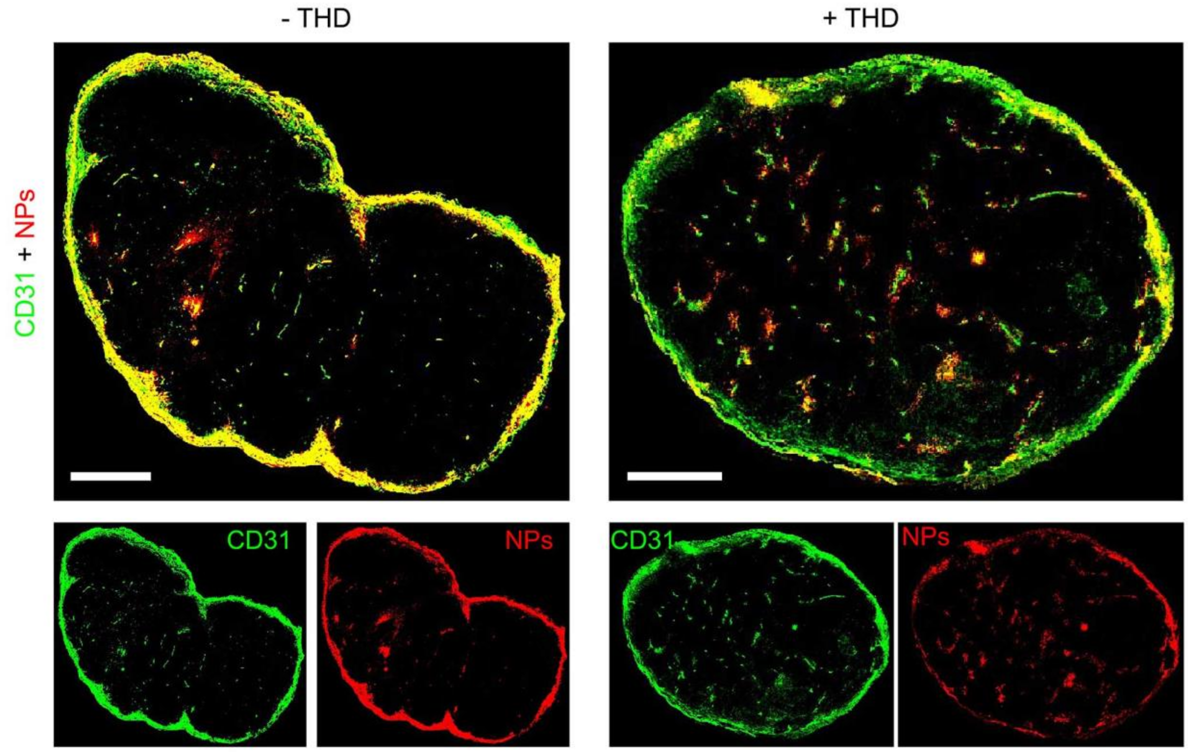

Figure S18. The vessel density and AAPS NPs distribution in HO-8910 tumor with or without THD treatments. HO-8910 tumor-bearing mice were injected intravenously with AAPS micelles. For thalidomide (THD) regulation groups, the mice were orally treated with THD (50 mg kg^{-1}) for three consecutive days before micelles administration. The tumors were excised at 24 h post-injection of micelles, sliced and stained with anti-CD31 antibody, finally scanned by a quantitative slide scanner. Tumor vessel and NPs were denoted as green and red signals, respectively. Scale bar = 500 μm.

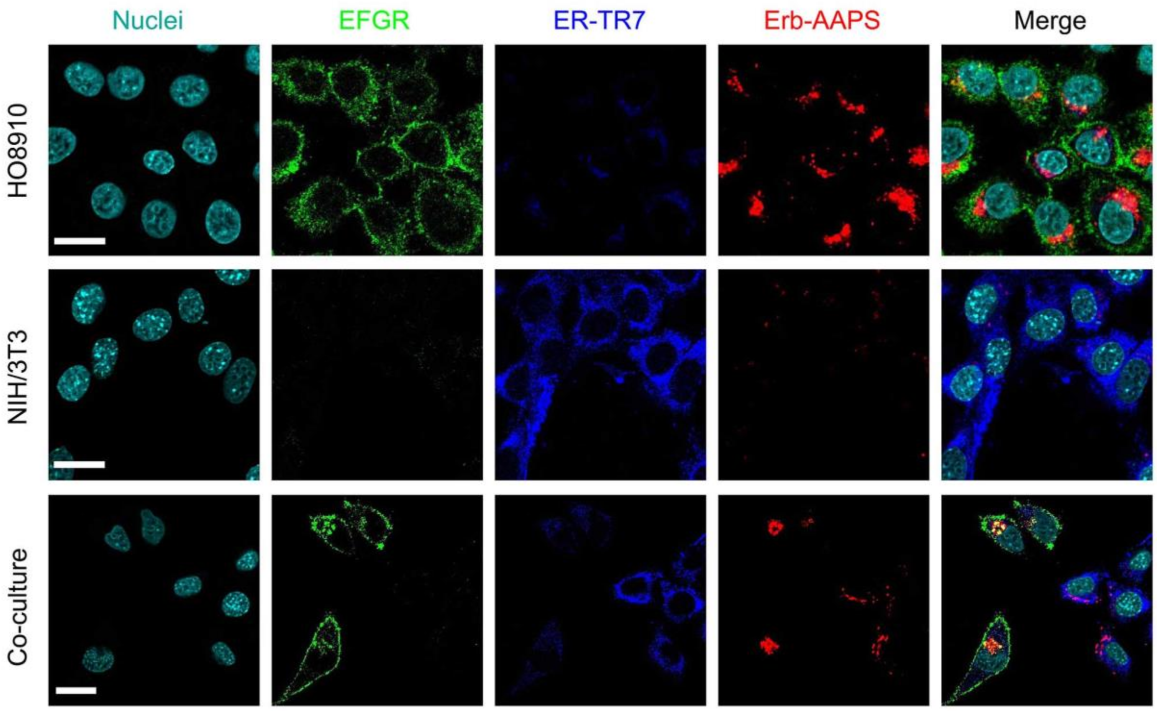

Figure S19. The comparison of cellular uptake between normal NIH/3T3 fibroblasts and CAFs. The NIH/3T3 fibroblasts and HO-8910 tumor cells were cocultured at 37 °C for 48 h to stimulate the cancer associated fibroblasts (CAFs) *in vitro*. Rabbit anti-EGFR-AF488 and rat anti-ER-TR7-PE antibodies were used to stain the HO-8910 and NIH/3T3 cells, and showed in green and blue signals, respectively. Erb-AAPS was showed in red signals. Scale bar = 20 μm.

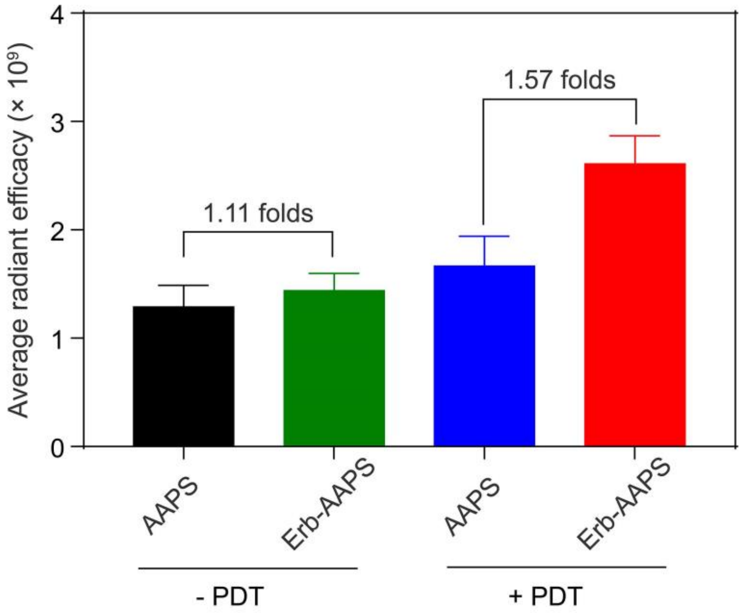

Figure S20. The quantitative intratumoral accumulation of micelles in HO-8910 tumors with or without pre-PDT treatments. Data were presented as mean ± s.d. (*n* =3).

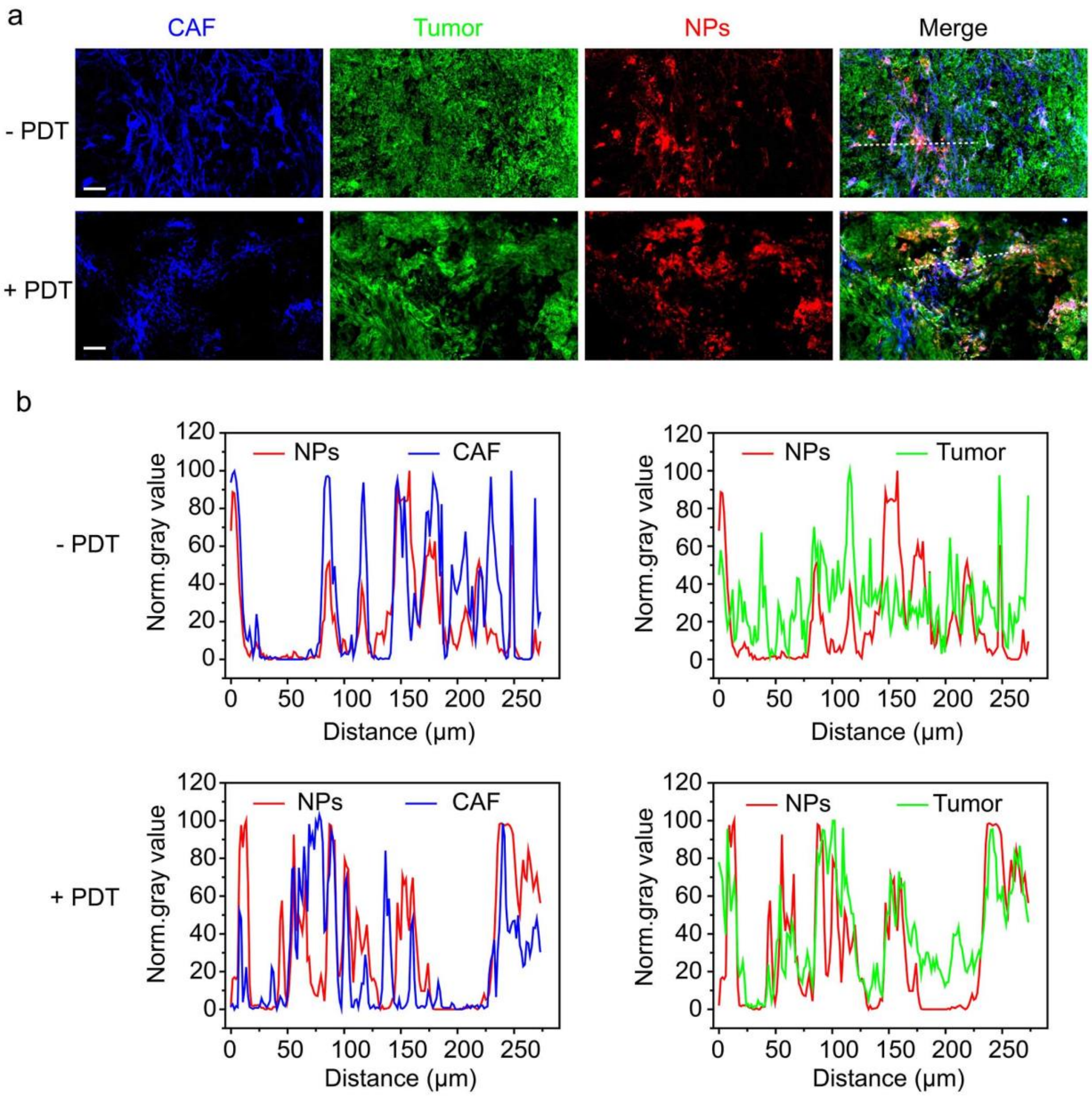

Figure S21. The colocalization analysis of Erb-AAPS NPs with tumor cells and CAFs. (a) The distributions of CAFs, tumor cells and Erb-AAPS in HO-8910 tumors with or without pre-PDT treatment. CAF, HO-8910 and NPs were denoted as blue, green, and red signals, respectively. Scale bar = 50 μm. (b) The colocalization between Erb-AAPS NPs and tumor cells (or CAFs) along the white dotted line in tumor slices of Fig.a. The data were analyzed by the Image J software. Norm.: Normalized.

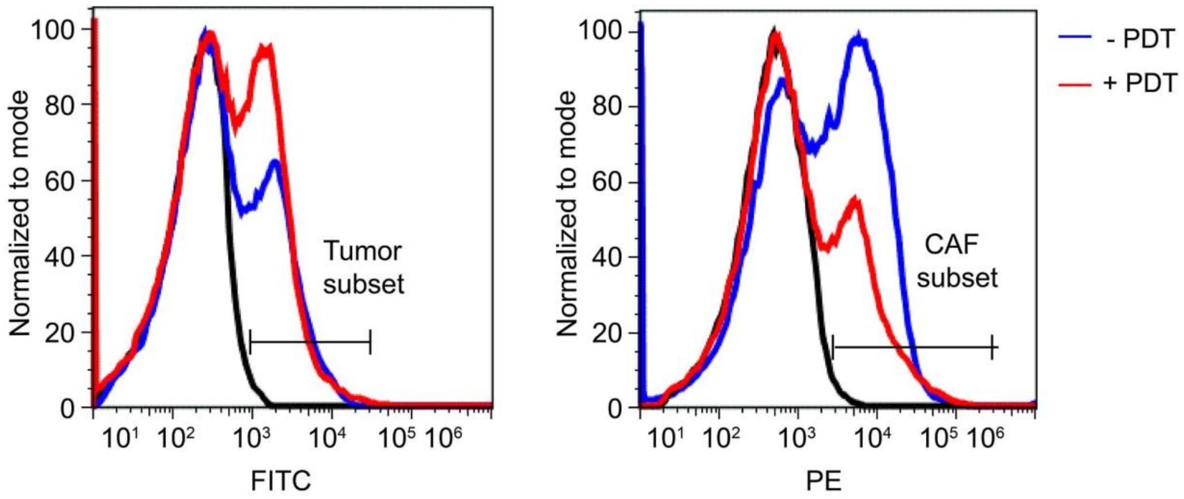

Figure S22. Quantitative of tumor cells and CAFs before or after pre-PDT treatment. The HO-8910 tumors with or without pre-PDT treatments were excised and digested to obtain single-cell suspension. Then HO-8910 tumor cells and cancer associated fibroblasts (CAFs) were immuno-labeled using rabbit anti-EGFR-AF488 and rat anti-ER-TR7-PE antibodies for FCM analyses, respectively.

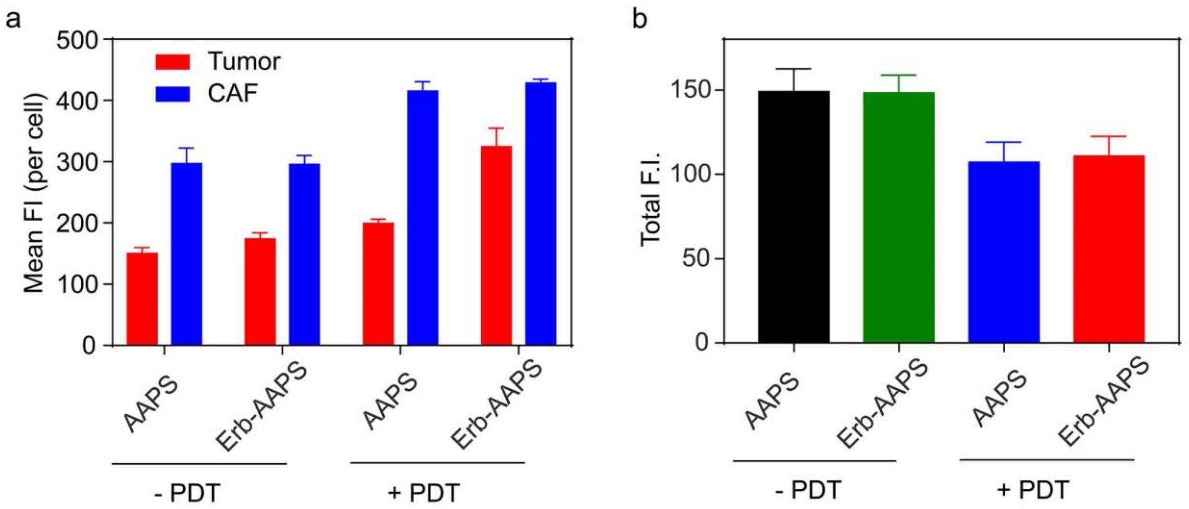

Figure S23. The *in vivo* cellular internalization of Erb-AAPS micelles after pre-PDT treatment. (a) FCM quantitative results for the mean fluorescence intensity (per cell) changes of AAPS and Erb-AAPS micelles that endocytosed by tumor cells or CAFs after pre-PDT treatment, respectively (n = 3). (b) The quantitative results for the total fluorescence intensity (per cell population) changes of AAPS and Erb-AAPS micelles endocytosed by CAFs after pre-PDT treatment (n = 3). All data were presented as mean ± s.d.

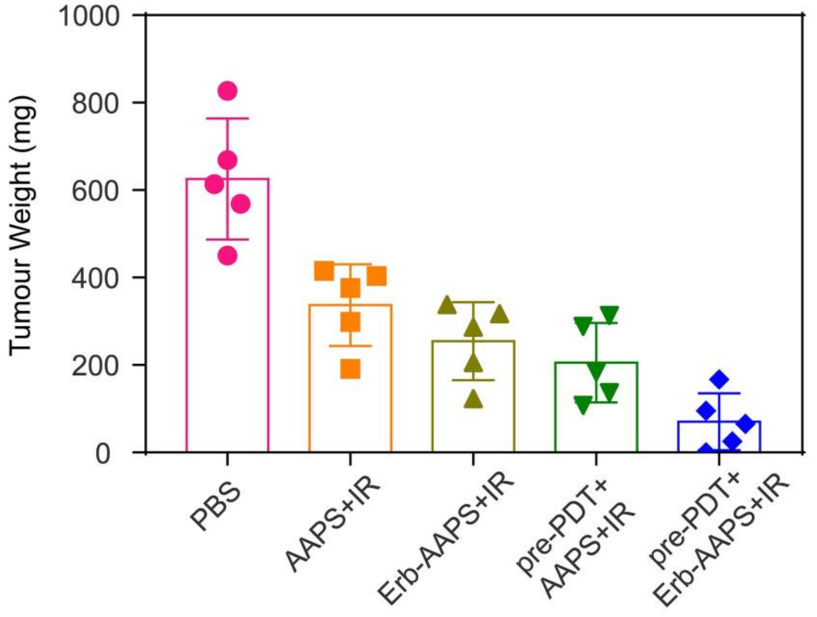

Figure S24. The weights of the excited tumors at the end of anti-tumor study. Data were presented as mean ± s.d. (n = 5).

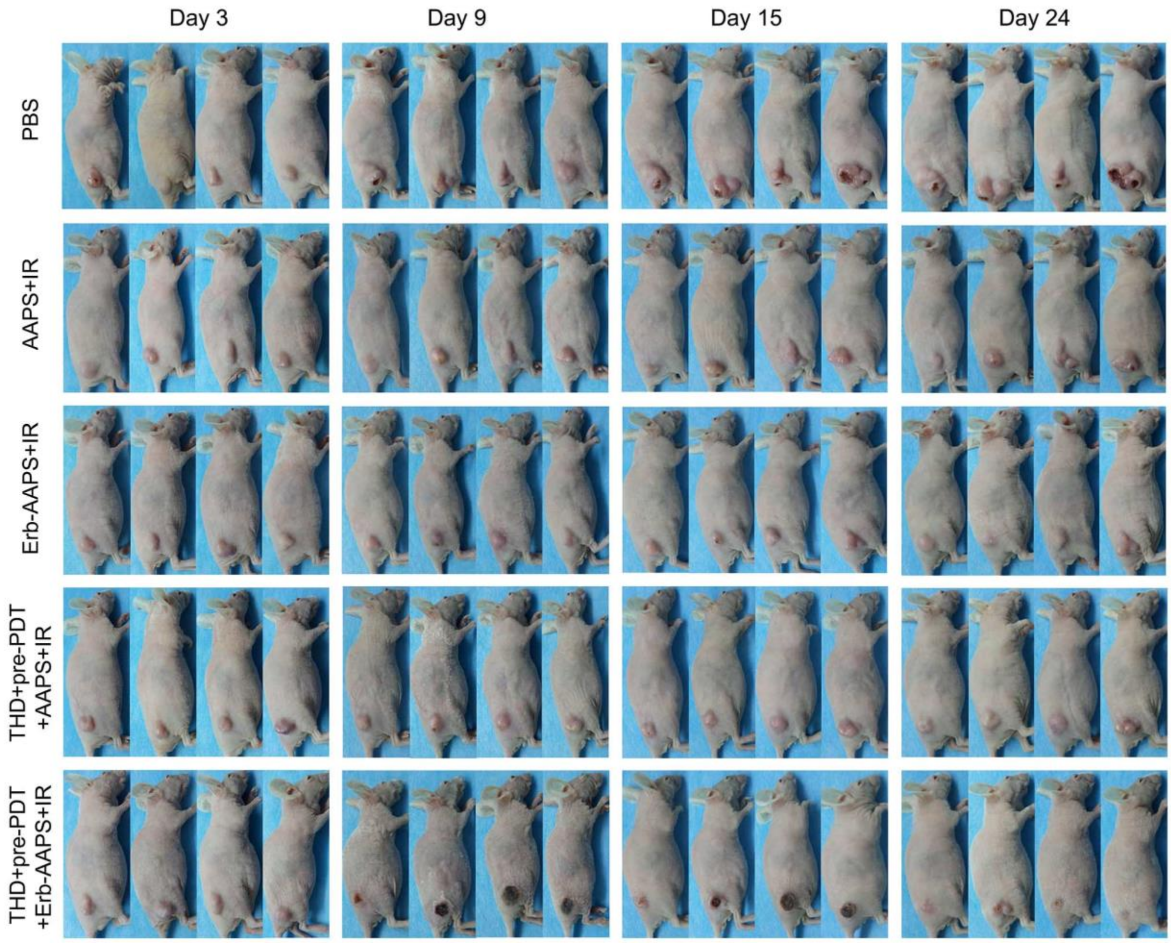

Figure S25. The photographic images of HO-8910 tumor-bearing mice during the anti-tumor study.

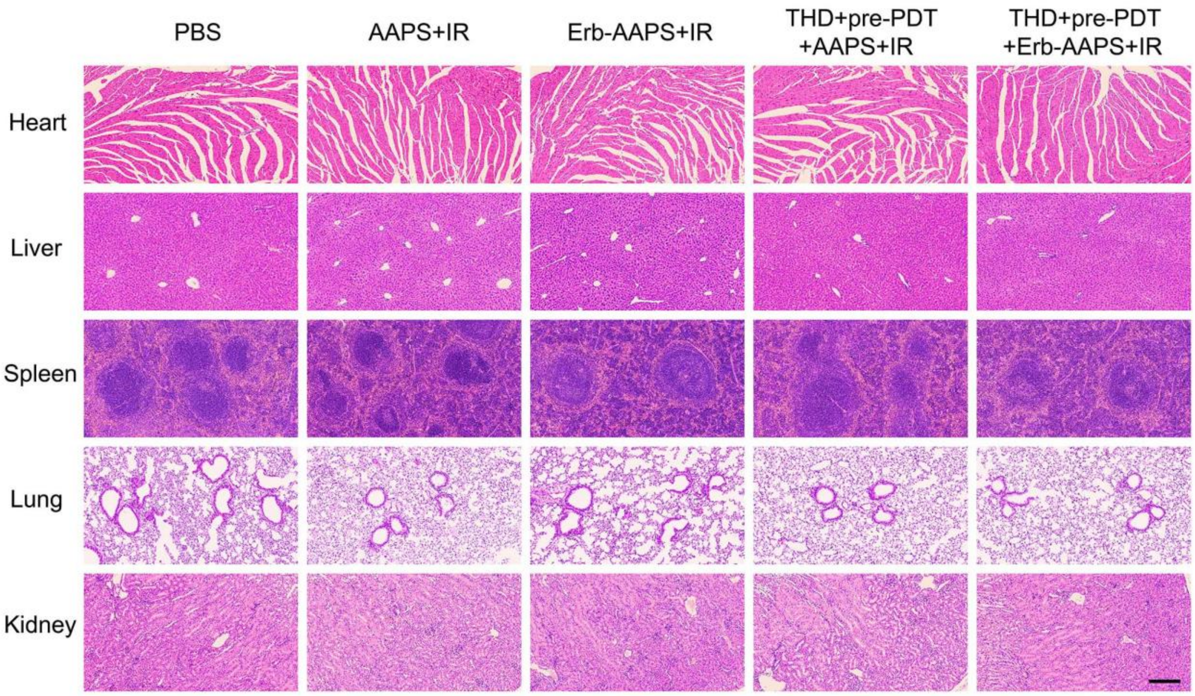

Figure S26. H&E staining of the major organs collected from HO-8910 tumor-bearing mice at day 30 of the anti-tumor study. Scale bar = 250 μm.

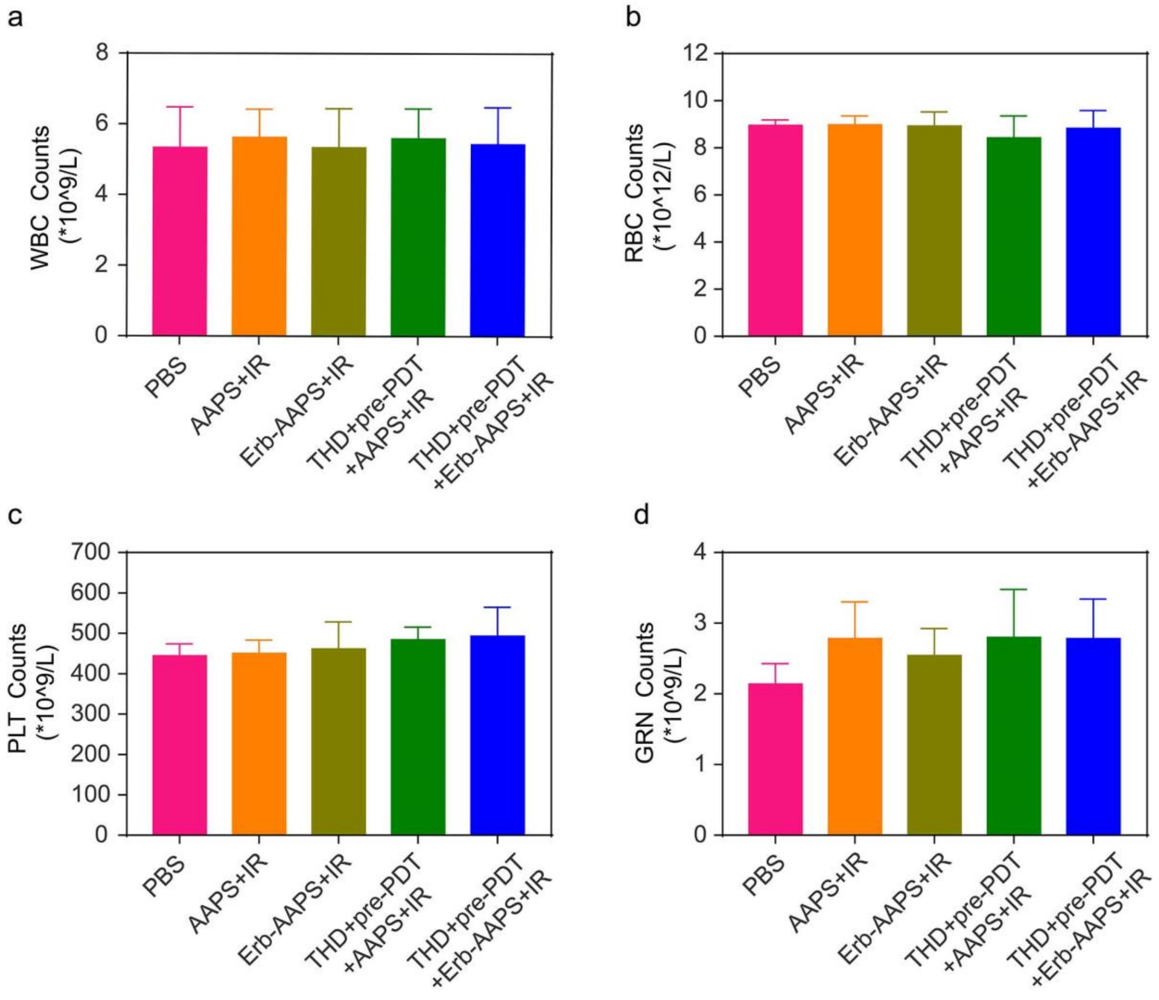

Figure S27. Blood routine examination of HO-8910 tumor-bearing mice at 24 h post PDT treatment. The (a) white blood cell (WBC), (b) red blood cell (RBC), (c) blood platelet (PLT) and (d) neutrophile granulocyte (GRN) counts of mice after different treatments. Data were presented as mean ± s.d. (n = 5).

References

[1] X. Ma, Y. Wang, T. Zhao, Y. Li, L. C. Su, Z. Wang, G. Huang, B. D. Sumer, J. Gao, *Journal of the American Chemical Society* **2014**, 136, 11085.

[2] Z. Valedkarimi, H. Nasiri, L. Aghebati-Maleki, J. Abdolalizadeh, M. Esparvarinha, J. Majidi, *Human antibodies* **2018**, 26, 171.

[3] K. Zhou, Y. Wang, X. Huang, K. Luby-Phelps, B. D. Sumer, J. Gao, *Angewandte Chemie* **2011**, 50, 6109.

[4] a) C. Mendoza, A. Desert, L. Khrouz, C. A. Paez, S. Parola, B. Heinrichs, *Environmental science and pollution research international* **2019**, DOI: 10.1007/s11356-019-04763-5; b) J. Shen, Z. J. Li, Z. F. Hang, S. F. Xu, Q. Q. Liu, H. Tang, X. W. Zhao, *Journal of nanoscience and nanotechnology* **2020**, 20, 3478; c) C. Mendoza, N. Emmanuel, C. A. Páez, L. Dreesen, J.-C. M. Monbaliu, B. Heinrichs, *ChemPhotoChem* **2018**, 2, 890.

[5] a) T. Qi, B. Chen, Z. Wang, H. Du, D. Liu, Q. Yin, B. Liu, Q. Zhang, Y. Wang, *Biomaterials* **2019**, 213, 119219; b) D. Liu, B. Chen, Y. Mo, Z. Wang, T. Qi, Q. Zhang, Y. Wang, *Nano letters* **2019**, 19, 6964.

[6] B. Chen, W. Dai, D. Mei, T. Liu, S. Li, B. He, B. He, L. Yuan, H. Zhang, X. Wang, Q. Zhang, *Journal of controlled release : official journal of the Controlled Release Society* **2016**, 241, 68.
